# Juvenile Osprey Navigation during Trans-Oceanic Migration

**DOI:** 10.1371/journal.pone.0114557

**Published:** 2014-12-10

**Authors:** Travis W. Horton, Richard O. Bierregaard, Peyman Zawar-Reza, Richard N. Holdaway, Paul Sagar

**Affiliations:** 1 Department of Geological Science, University of Canterbury, Christchurch, New Zealand; 2 Biology Department, University of North Carolina at Charlotte, Charlotte, NC, United States of America; 3 Geography Department, University of Canterbury, Christchurch, New Zealand; 4 School of Biological Science, University of Canterbury, Christchurch, New Zealand; 5 National Institute of Water and Atmospheric Research, Christchurch, New Zealand; University of Pécs Medical School, Hungary

## Abstract

To compensate for drift, an animal migrating through air or sea must be able to navigate. Although some species of bird, fish, insect, mammal, and reptile are capable of drift compensation, our understanding of the spatial reference frame, and associated coordinate space, in which these navigational behaviors occur remains limited. Using high resolution satellite-monitored GPS track data, we show that juvenile ospreys (*Pandion haliaetus*) are capable of non-stop constant course movements over open ocean spanning distances in excess of 1500 km despite the perturbing effects of winds and the lack of obvious landmarks. These results are best explained by extreme navigational precision in an exogenous spatio-temporal reference frame, such as positional orientation relative to Earth's magnetic field and pacing relative to an exogenous mechanism of keeping time. Given the age (<1 year-old) of these birds and knowledge of their hatching site locations, we were able to transform Enhanced Magnetic Model coordinate locations such that the origin of the magnetic coordinate space corresponded with each bird's nest. Our analyses show that trans-oceanic juvenile osprey movements are consistent with bicoordinate positional orientation in transformed magnetic coordinate or geographic space. Through integration of movement and meteorological data, we propose a new theoretical framework, chord and clock navigation, capable of explaining the precise spatial orientation and temporal pacing performed by juvenile ospreys during their long-distance migrations over open ocean.

## Introduction

How do animals navigate during long-distance migration? Finding an answer to this complex problem requires an integrated research approach involving scientists from a variety of disciplines, and any attempt at an answer must start by documenting what animals do during migratory movement. The widespread application of high-resolution satellite-monitored platform transmitter terminal (PTT) technology creates an unprecedented opportunity to explore how animals achieve their remarkable long-distance migrations.

Of all the movement behaviors exhibited by migrating birds, perhaps the most remarkable is also the most common: drift compensation. Although displacement by wind complicates a bird's ability to successfully navigate between habitats, several studies have demonstrated that many birds, including ospreys [1], European honey buzzards (*Pernis apivorus*) [1], western marsh harriers (*Circus aeruginosus*) [2], passerines (e.g. *Phylloscopus trochilus*)[3], [4], near passerines (e.g. *Merops apiaster*) [5], and a variety of shorebirds (e.g. *Calidris canutus*; *Limosa lapponica*; *Pluvialis squatarola*; *Charadrias hiaticula*; *Arenaria interpres*; *Calidris alba*) [6], have the ability to compensate for the effects of wind drift. Many other animals are also capable of current compensation, including: marine mammals [7], reptiles [8], fish [9], and insects [10]. Yet, a fundamental question remains largely unanswered despite the widespread nature of these findings: how do they do it?

Although a variety of theoretical frameworks capable of explaining how animals might navigate have been published [11]–[14], the majority are based on experimental results determined under controlled conditions. It remains to be determined which of these theoretical frameworks are capable of explaining the movement behaviors of free-ranging wild animals. Advances in animal tracking technology, meteorological monitoring, magnetic modelling, and computer processing power create opportunities to address this gap in our understanding of movement behavior. Although significant progress has been made, conflicting interpretations [15] and the complexity of studying animal movement behavior has left the mechanisms of animal navigation as one of the most enduring mysteries of biological science [16].

Experimental and empirical studies suggest that animals may navigate using one or more of the following mechanisms: vector orientation/path integration [17], sun compass orientation [18], celestial orientation [19], magnetic compass orientation [20], magnetic map orientation [21], olfactory homing [22], and infrasonic orientation [23]. Recent reviews [24], [25] indicate that it is almost certain that most, if not all, animals utilize more than one of these approaches when solving the challenges of orientation and navigation. The observation that adult European ospreys are capable of >70% wind drift compensation while juveniles experience full wind drift suggests navigational behavior may even be age dependent [1].

However, the observation that juvenile ospreys migrating over land do not compensate for wind drift does not demonstrate that they are incapable of wind drift compensation. To explore this possibility, we present an integrated analysis of geospatial, meteorological, and magnetic coordinate data derived from high resolution, satellite-monitored global positioning system (GPS) track data determined for ten <1 year-old ospreys migrating across the western Atlantic Ocean. In this study we first quantitatively describe the constant course geometry of the birds' movements. Second, we perform a spatio-temporal wind drift analysis of the birds' tracks by integrating the geospatial results with dynamic data-based regional mesoscale meteorological modelling to test the hypothesis: juvenile ospreys experience full wind drift during long-distance migration over open ocean. Third, we explore the possible mechanisms of osprey positional orientation through analysis of Enhanced Magnetic Model coordinate data. We conclude by presenting a new theoretical framework of animal navigation that is compatible with all of the empirical results we report.

## Materials and Methods

### Tag Deployment

Ten fully flighted juvenile ospreys were captured on their nests between one and three weeks post-fledging using a noose carpet baited with fish [26]. 30-g solar-powered Argos/GPS transmitters (model PTT-100; Microwave Telemetry, Inc.) were attached to the birds using 7 mm wide Teflon ribbon in a backpack arrangement [27]. The UNC-Charlotte Institutional Animal Care and Use Committee approved this research (IACUC approval number: 08-024).

### High Resolution Tracking

**Figure 1:**
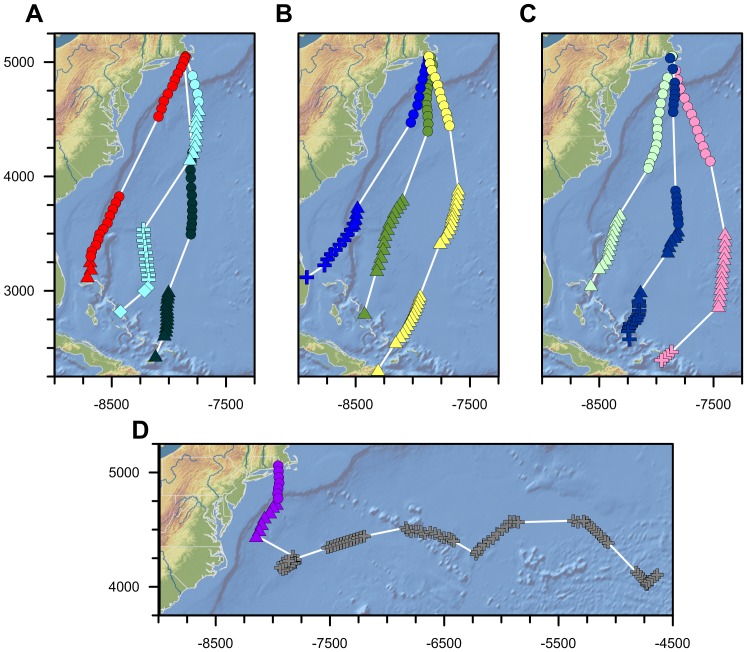
Trans-oceanic juvenile osprey migration track maps (Mercator Projection). Colors correspond with individual ospreys (pink  =  Belle; red  =  Felix; yellow  =  Moffet; light green  =  Henrietta; green  =  Bea; dark green  =  Luke; light blue  =  Caley; royal blue  =  Mittark; dark blue  =  Isabel; purple/gray  =  Chip). Symbols correspond with different constant course track segments identified by piecewise linear regression breakpoint analysis (circles  =  first track segment following departure; triangles  =  second track segment; addition symbols  =  third track segment; diamonds  =  fourth track segment). Only the trans-oceanic portion of each bird's migration is shown. Gray addition symbols correspond with Chip's movements following his first night aloft, presumably when he was resting on or in contact with one or more vessels (see text). Northing and Easting values are shown in kilometers.

The GPS-enabled PTT tags we deployed are accurate to ±18 m with temporal resolutions as fine as 45 seconds. In an effort to maximize transmitter longevity, the rechargeable solar powered GPS PTT data we report were recorded at consecutive 1-hour intervals across a 12-hour period each day. This duty-cycle was chosen to conserve battery power and because it was assumed that the birds would roost during hours of darkness. The majority of tracked birds migrated largely over land or coastal environments, making it impossible to differentiate true navigational behavior from piloting behavior (i.e. Griffin's Type III navigation from Type I visual landmark-based navigation) [28], based on track data alone (Figure S1). However, ten of the twenty-four juveniles tagged in New England performed unexpected non-stop migratory movements over the western Atlantic Ocean (Figure 1), thereby presenting an opportunity to study the navigational behaviors of these birds in the absence of visual landmarks. These migrations over open ocean were surprising because Ospreys are terrestrial birds and cannot land on water. Nine of the juveniles birds we studied were tagged on Martha's Vineyard, Massachusetts, and the tenth was tagged in central New Hampshire, U.S.A. (Table 1).

**Table 1:** Summary of juvenile osprey tagging locations and constant-course migration segments.

Osprey (segment)	Date of first transmission	Date of last transmission	Start Latitude	Start Longitude	End Latitude	End Longitude	n	Distance Traveled (km)	Duration (hours)	Average Velocity (km/h)	Mean Track Segment Direction (°)	Straightness Index
Bea (tagging)	8/5/2009		41.3873	−70.4707								
Bea (1)	9/14/09 1:00 PM	9/15/09 1:00 AM	41.3525	−70.4550	36.8677	−70.6928	13	507.3	12.0	42.2 (±8.7)	184.9 (±9.9)	0.9839
Bea (2)	9/15/09 1:00 PM	9/16/09 1:00 PM	32.4588	−72.6775	24.6273	−75.6693	14	925.2	19.3	47.8 (±8.7)	200.8 (±9.2)	0.9927
Belle (tagging)	7/28/2010		41.4692	−70.6235								
Belle (1)	10/16/10 1:00 PM	10/17/10 12:00 AM	41.4342	−70.6035	34.9578	−67.6265	12	674.2	11.0	61.2 (±4.3)	160.3 (±6.0)	0.9950
Belle (2)	10/17/10 12:00 PM	10/18/10 12:00 AM	30.1502	−66.4967	25.0915	−66.9767	13	566.1	12.1	46.9 (±4.1)	184.9 (±4.4)	0.9972
Belle (3)	10/18/10 12:00 PM	10/18/10 3:00 PM	21.7278	−70.6900	21.1202	−71.4668	4	105.2	3.0	35.0 (±5.1)	229.8 (±3.8)	0.9986
Caley (tagging)	8/3/2009		41.3732	−70.5027								
Caley (1)	9/19/09 1:00 PM	9/19/09 4:00 PM	41.3758	−70.5095	38.7020	−69.5030	4	180.3	3.0	60.0 (±4.4)	165.9 (±3.0)	0.9992
Caley (2)	9/19/09 5:00 PM	9/20/09 1:00 AM	38.2512	−69.5047	35.0937	−70.2163	9	361.9	8.2	44.0 (±3.1)	189.9 (±10.1)	0.9858
Caley (3)	9/20/09 1:00 PM	9/20/09 10:00 PM	30.3983	−73.8658	26.9340	−73.4510	10	388.0	9.0	43.1 (±4.4)	174.0 (±3.7)	0.9982
Caley (4)	9/21/09 12:00 AM	9/21/09 1:00 PM	26.3837	−73.5220	24.6775	−75.6710	3	287.3	8.2	35.1 (±1.2)	227.1 (±3.1)	0.9996
Felix (tagging)	8/16/2007		41.5783	−70.6168								
Felix (1)	9/16/07 1:00 PM	9/17/07 8:00 PM	41.3398	−70.6032	28.5815	−78.0095	21	1572.1	31.0	50.6 (±6.3)	203.7 (±7.3)	0.9983
Felix (2)	9/17/07 9:00 PM	9/18/07 1:00 PM	28.2437	−78.0543	27.1933	−78.2622	3	122.3	2.3	53.3 (±14.5)	188.7 (±20.3)	0.9692
Henrietta (tagging)	5/24/2011		41.4667	−70.5673								
Henrietta (1)	9/16/11 1:00 AM	9/17/11 1:00 AM	41.4625	−70.6238	34.5040	−72.4998	14	1008.0	18.7	54.0 (±17.6)	192.3 (±7.1)	0.9941
Henrietta (2)	9/17/11 1:00 PM	9/18/11 1:00 PM	31.4662	−74.7465	26.5962	−77.0080	14	674.5	18.4	36.7 (±5.1)	202.1 (±9.8)	0.9882
Isabel (tagging)	8/1/2009		41.4672	−70.5662								
Isabel (1)	9/5/09 4:00 PM	9/6/09 7:00 PM	41.3443	−70.7728	30.7875	−70.1300	15	1179.7	24.9	47.3 (±17.6)	178.4 (±6.3)	0.9963
Isabel (2)	9/6/09 8:00 PM	9/7/09 3:00 PM	30.3745	−70.1795	25.6753	−73.2575	8	611.9	15.5	39.5 (±10.6)	206.3 (±13.6)	0.9863
Isabel (3)	9/7/09 4:00 PM	9/8/09 1:00 PM	25.3978	−73.2572	22.6772	−73.9887	11	339.0	11.1	30.6 (±5.2)	198.4 (±24.5)	0.9190
Luke (tagging)	7/27/2007		41.4750	−70.6125								
Luke (1)	11/8/07 11:00 AM	11/8/07 10:00 PM	41.2717	−70.66933	30.0965	−70.1470	13	1246.5	24.0	51.9 (±10.6)	181.4 (±3.8)	0.9979
Luke (2)	11/9/07 10:00 AM	11/10/07 10:00 AM	26.1322	−71.8860	21.4823	−72.9247	14	383.6	13.1	29.2 (±3.5)	186.8 (±8.5)	0.9926
Mittark (tagging)	9/16/2008		41.4025	−70.68233								
Mittark (1)	10/5/08 3:00 PM	10/6/08 12:00 AM	41.36383	−70.65233	37.4155	−72.0177	10	588.4	10.7	55.0 (±9.3)	194.5 (±4.4)	0.9971
Mittark (2)	10/6/08 2:00 PM	10/6/08 5:00 PM	31.9525	−76.22067	30.8112	−76.4960	4	151.4	3.5	43.2 (±1.7)	194.8 (±6.7)	0.9988
Mittark (3)	10/6/08 6:00 PM	10/7/08 3:00 PM	30.39383	−76.70983	27.1190	−80.19483	9	635.7	13.1	48.6 (±1.8)	219.4 (±9.5)	0.9800
Moffet (tagging)	8/4/2009		41.4157	−70.5637								
Moffet (1)	10/8/09 3:00 PM	10/9/09 12:00 AM	41.4305	−70.5928	37.2007	−68.9812	11	491.8	10.0	49.1 (±3.3)	164.4 (±5.0)	0.9970
Moffet (2)	10/9/09 1:00 AM	10/11/09 1:00 PM	33.1452	−68.2823	20.4677	−74.5888	27	1558.0	43.9	35.4 (±6.6)	202.6 (±8.6)	0.9894
Chip (tagging)	8/2/2012		43.4577	−71.5682								
Chip (1)	10/7/12 1:00 AM	10/7/12 5:00 PM	41.4978	−71.4515	39.8313	−71.4847	6	186.2	5.0	37.2 (±2.6)	179.4 (±6.8)	0.9952
Chip (2)	10/7/12 6:00 PM	10/8/12 1:00 AM	39.5482	−71.4317	37.2432	−73.1692	8	302.8	7.0	43.2 (±6.5)	211.0 (±12.0)	0.9831
						**Average (±1σ)**	**11 (±6)**	**601.9 (±425.6)**	**13.5 (±9.6)**	**44.8 (±8.6)**	**192.9 (±18.0)**	**0.9893 (±0.0164)**

Variance in average velocity and mean track segment direction is reported as ±1σ.

### Wind Vector Analysis

Wind directions and speeds at each bird location were determined using a dynamic data-based regional mesoscale meteorological model [29]. The wind field was simulated using the Weather Research and Forecasting model (WRF, version 3.5), which is the state-of-the-art code and is used by many operational and research institutes to study and forecast weather [30]. For this study, WRF simulated weather for a single geographic domain at 30 km resolution with a north-south extent of 7920 km, and an east-west extent of 3960 km. The meteorological initial and boundary conditions come from the National Centers for Environmental Prediction (NCEP) Final Analysis (FNL) re-analysis data at 1° resolution (http://www.nomad3.ncep.noaa.gov/ncepdata). Re-analysis was used at 6-hourly intervals to force the model to take into account and be compliant with the evolving and propagating synoptic weather patterns (i.e. the high and low pressure systems). For the purpose of this study we used the computed east-west and north-south components of wind velocity at 10 m altitude (U10 and V10, respectively). The lower-most layers of the troposphere are well-mixed over the oceans, thus departures from the wind velocity field at 10 m is negligible for the first 500 m. The average flying altitude of the 10 osprey tracks we report was 264 meters (±224 m, 1σ).

**Figure 2:**
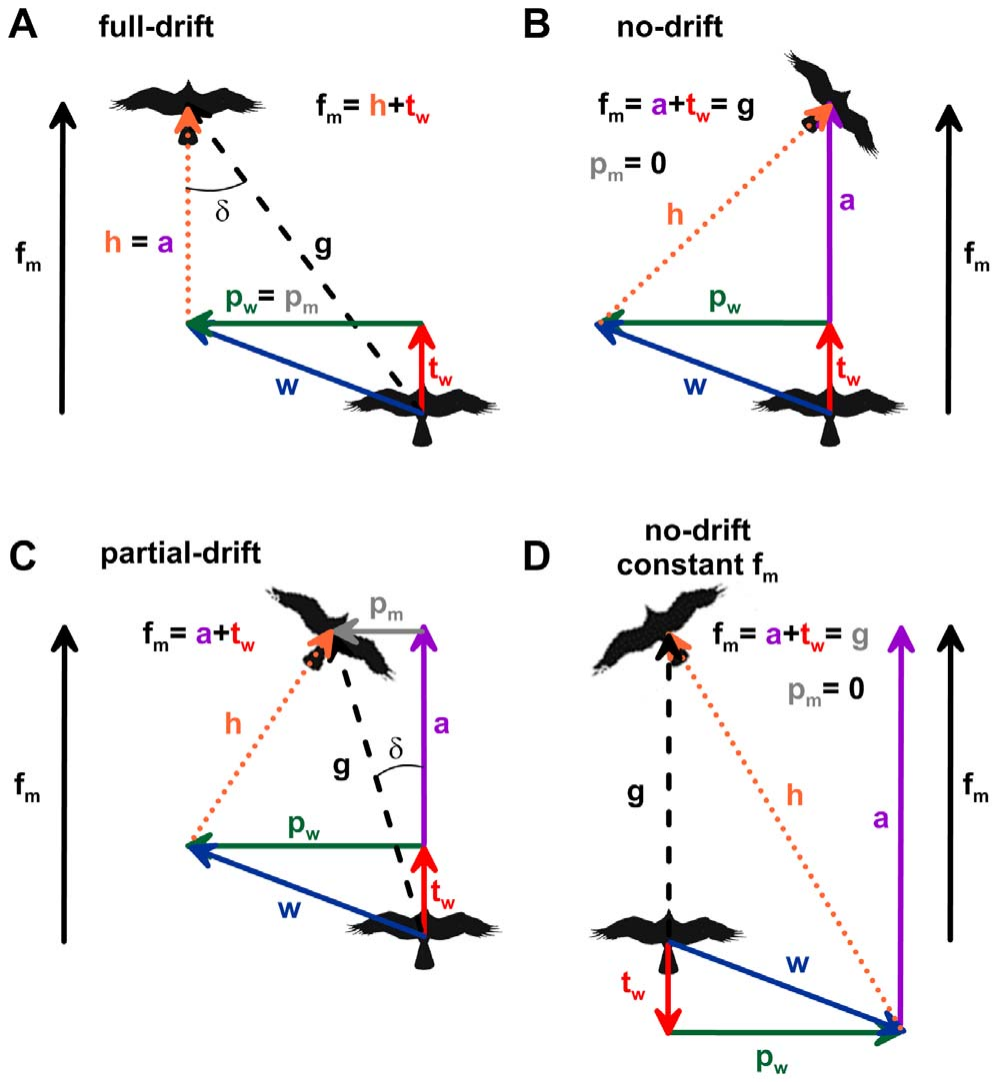
Vector representations of various movement behaviors in response to wind. Animals moving through air and water exhibit a variety of orientational responses to flow [31], including full drift (A), no drift (B), partial drift (C), and full compensation of both the perpendicular and parallel wind drift vectors (D). In these diagrams, the groundtrack direction and speed (g) determined from sequential PTT tag locations represents the vector sum of the wind vector (w) and the bird's heading vector (h). These three vectors represent the classic ‘wind triangle’. Wind drift analysis requires further determination of components of these vectors relative to a fixed direction, here defined as the mean track segment direction. The direction of the forward movement (f_m_) vector equals the mean track segment direction and the forward movement velocity equals the groundspeed times the cosine of the drift angle (δ), where, δ =  groundtrack direction - mean track segment direction. The forward movement vector must also equal the tailwind vector (t_w_) plus the active forward movement vector (a). The perpendicular wind (p_w_) and perpendicular movement (p_m_) vectors are the vector components of the wind vector and groundtrack vector, respectively, that are perpendicular to the mean track segment direction. In order to maintain a constant course movement in dynamic flows a migrating animal must be able to monitor and adjust the magnitude and direction of its heading vector at regular intervals that are smaller than the duration of the constant course movement.

As part of our wind analysis, we quantified several different vectors relevant to movement behavior (Figure 2). These vectors, determined between individual bird locations, include: the groundtrack vector derived from the PTT tag data (g), the modelled wind vector (w), the heading ‘airspeed’ vector (h), the perpendicular wind vector (p_w_), the tailwind vector (t_w_), the perpendicular bird movement vector (p_m_), the forward movement vector (f_m_), and the active forward movement vector (a_m_). The groundtrack vector, wind vector, and the heading vector represent the classic wind triangle, wherein the observed groundtrack vector represents the sum of the wind and heading vectors. In contrast, the perpendicular wind and perpendicular bird movement vectors, as well as the tailwind, forward movement and active forward movement vectors, were all calculated relative to the observed mean track segment direction (Figure 2), thus enabling an analysis of the effects of wind displacement at the hourly scale (i.e. the resolution of the PTT tag data) relative to individual track segment directions.

The track segment approach we apply is the only way to explore each bird's individual movement behaviours. This new individual approach to intra-track analysis represents an important step forward in animal navigation research, as both the track data we report and the osprey migration track data published by others [32] clearly demonstrate that juvenile osprey migratory directions and destinations are highly individual (Figure 1; Table 1). Such behavioral variability in a population is to be expected [33], and intensive study of movement behaviors at the individual level is likely to lead to new and unexpected insights [34]. We highlight that such insights cannot be gained if we assume all birds from a given population share the same preferred migratory direction or destination, or both.

### Magnetic Coordinates

**Figure 3:**
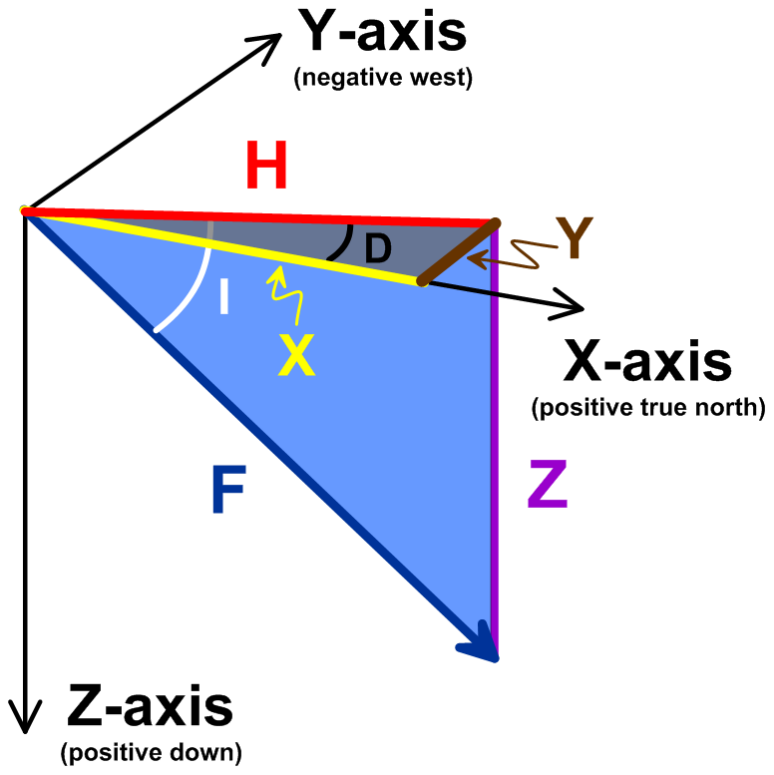
The Constantly Changing Seven Magnetic Field Elements. The magnetic field experienced at or near the Earth's surface is constantly changing in both space and time due to secular variation in the Main Field (>90% of the total field intensity), interactions with the unpredictable and temporally dynamic interplanetary magnetic field (largely of solar origin and as large as 10% of the total field intensity) and the crustal anomaly field (<1% of the total field intensity). The seven elements include: F, the total field intensity measured in nanotesla (SI) or gauss (CGS); D, the declination angle measured positive to the east of true north; I, the inclination angle measured positive in the downward direction relative to horizontal; H, the horizontal component of the total field intensity; X, the north-south component of H measured positive to the north; Y, the east-west component of H measured positive to the east; Z, the vertical component of the total field intensity measured positive in the downward direction. X, Y, and Z define a three-dimensional Cartesian coordinate space, whereas F, I, and D define a three-dimensional spherical coordinate space. The bicoordinate F-I space is polar by definition and is equivalent to the two-dimensional H-Z Cartesian coordinate space (see also Figure 7).

We determined spherical (magnetic field intensity, F; inclination, I; declination, D) and Cartesian (true north-south, X; east-west, Y; vertical, Z; horizontal, H) magnetic field coordinates (Figure 3) at each bird location using the Enhanced Magnetic Model (EMM) [35]. The EMM differs from the World Magnetic Model (WMM) in that it integrates both the Earth's main magnetic field as well as its crustal anomaly field. The inclusion of magnetic anomalies associated with rocks and minerals was not possible prior to GFZ-Potsdam's CHAMP satellite mission, in addition to a global compilation of aeromagnetic, marine, and continental magnetic survey data [35]. The free and downloadable 2010–2015 epoch EMM was released in late 2006 and was extended backwards in time to include the 2000–2005 and 2005–2010 epochs in early 2012 (available at http://ngdc.noaa.gov/geomag/EMM).

Another novel aspect of our magnetic field analysis is the temporal context. Using the EMM, we were able to determine the magnetic field coordinates at each bird's nesting site during the incubation period. This knowledge allows us to transform the magnetic coordinate data for each bird's track such that the origin of the magnetic coordinate space corresponds with each bird's nesting site, rather than the subcrustal origin of the magnetic field assumed by the EMM and all other geomagnetic models. We used a series of sequential vector coordinate transformations to achieve this change in origin. First, the magnetic coordinate space was horizontally rotated about the vertical axis by the magnetic declination at the nesting site according to:
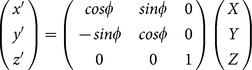



where, *x’, y’, z’* are the horizontally-rotated transformed Cartesian magnetic coordinates, φ is the nesting site magnetic declination in degrees, and *X, Y, Z* are the Cartesian magnetic coordinates at the bird's location generated by the EMM. Second, the horizontally-rotated magnetic coordinates were vertically-rotated about the new y’ horizontal axis by the magnetic inclination at the nesting site according to:
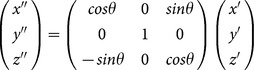



where, *x”, y”, z”* are the vertically and horizontally rotated transformed Cartesian magnetic coordinates, and θ is the nesting site magnetic inclination in degrees. Third, the vertically and horizontally rotated transformed coordinates were translated along the x” axis by the magnetic field flux density (*F*) at the nesting site according to:
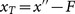


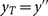


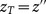



where, *x_T_, y_T_, z_T_* are the fully transformed Cartesian magnetic coordinates relative to a nesting site coordinate space origin (i.e. {*x_T_, y_T_, z_T_}  = * {0, 0, 0} at each bird's nesting site). Such transformations facilitate analysis of animal movements in a magnetic coordinate space that is arguably more meaningful to the animal: a magnetic coordinate space relative to its natal home. Birds, and other terrestrial oviparous animals, are particularly attractive candidates for this type of analysis as the egg-laying site and animal age are more easily constrained than is possible for viviparous and marine oviparous animals such as marine mammals and fish.

### Statistical Analyses

We used a variety of statistical methods to analyse our data. The trans-oceanic migration portion of each bird's movements was separated into sequential track segments using piecewise linear regression breakpoint analysis (PLR-BPA) performed on Mercator coordinate data using the *segmented* package in [R] [36]; *segmented* estimates generalized linear models for a bivariate dataset using an iterative approach based on a fixed number of breakpoints determined using the breakpoint command in *segmented*. The straightness of each PLR-BPA track segment was then determined using Batschelet's straightness index (i.e. the ratio between the rhumb-line distance and the actual distance travelled between the first and last locations for each segment) [37]. We restricted these geospatial analyses to the open ocean movements for each bird (Figure 1).

We analysed our wind vector data using two different significance tests. First, the statistical significance of the linear relationships between perpendicular movement and perpendicular wind velocities, forward movement and tailwind velocity, and active forward movement velocity and tailwind velocity were tested using a linear model analysis of variance (ANOVA) in [R]. Second, the significance of the differences between mean bird headings and mean wind vector directions, and mean bird headings and mean bird ground track bearings were tested using the Watson two-sample test of homogeneity in the [R] CircStats package [38]. CircStats was also used to determine the mean values and 95% confidence intervals of the wind direction, bird heading, and bird ground track bearing datasets (bootstrapped von Mises distribution with 1000 re-samplings of each dataset).

We also tested the significance of the difference between regression equation coefficients (e.g. slope of a linear regression) of all migration segments in each of three different bicoordinate spaces: Mercator (easting versus northing), geomagnetic (inclination versus intensity), and transformed magnetic (*y_T_* versus *z_T_*). These significant difference tests were performed by calculating the t-statistic for each pair of migration segment regression coefficients and comparing these values to the corresponding t-critical value at the appropriate degrees of freedom and an α = 0.05 significance level. Corresponding p-values were calculated from these parameters using a two-tailed t-distribution. Straightness index values [37] were also determined for each track segment in Cartesian transformed magnetic coordinate space.

## Results and Discussion

### Trans-Oceanic Movements of Juvenile Ospreys

The trans-oceanic movements of the juvenile ospreys we report are remarkable in three main ways. First, the arrival of nine of the ten juvenile ospreys tracked across the western Atlantic Ocean on Caribbean islands following 36–54 hours of non-stop flight, demonstrates that <1 year-old ospreys are capable of continuous long-distance migrations over expansive water bodies during both day and night despite the absence of visual landmarks, stop-over locations, and foraging habitats (Figure 1). Such long-distance trans-oceanic movements by naïve terrestrial birds are difficult to explain from ecological first principles. What compels these ospreys to set off across a body of water of unknown width and limited foraging potential rather than following the coast, as is notably practiced by other ospreys from the same region (Figure S1) and many other bird species [39]? Although the answer to this question is beyond the scope of this research, it highlights the difficulty in comprehending these trans-oceanic movements in the absence of a strong endogenous urge or exogenous cue to migrate.

Second, the duration and distance of these movements are long, particularly in the context of the birds' age. The average open ocean distance travelled by the nine birds making landfall in the Caribbean was 2162±326 km (±1σ) over an average time period of 52±12 hours (±1σ), equating to an average travelling velocity of 41.3 km/h. In contrast, the longest non-stop pre-migration flight performed by any of these birds was a 9.5 hour, 205 km (21.6 km/h average velocity), return flight to Martha's Vineyard on Sept. 4, 2010 performed by Belle, following a 3-day excursion in southern New Hampshire (Figure S2). None of the birds we tracked had flown more than 10 km during hours of darkness before departing the coast on their non-stop southward migrations. Thus, the tracking data we report indicate that during their southward migrations, these birds on average flew anywhere between 2 and 10 times as fast, as far, and as long as they ever had before.

Third, perhaps the most remarkable result of the osprey tracks we report is their straightness (Figure 1; Table 1). Not only are these birds flying faster, farther, and longer across an unfamiliar oceanic seascape, but they are also doing it in a series of near perfect constant course migratory segments. Twenty-three of the twenty-five track segments identified in our PLR-BPA of the individual trans-oceanic migration tracks exhibit straightness index values >0.98, and twenty-four of the twenty-five segments exhibit straightness index values of >0.95 (Table 1). The high resolution GPS track data reveal that every one of these birds demonstrated a navigational capacity to be no more than 10 km off course for every 1000 km travelled. In fact, the longest distance constant course track segment we identified (1572 km) was only 2.7 km longer than a perfectly straight rhumb line movement. For context, this movement is comparable in size to the rhumb line distance separating Washington D.C. and Miami, or Seattle and Los Angeles, with a navigational ‘error’ as small as Manhattan Island across its shortest axis.

Despite these remarkably precise movements, one of the birds (‘Chip’; Figure 1) did not make it to the Caribbean despite departing the coast along a similar trajectory to many of the other birds. Sometime in the middle of his first night aloft, Chip stopped making progress south. Time series analysis of Chip's ground speed and altitude on subsequent days indicate that he spent several days on, or following, one or more vessels from his second day at sea until his transmitter stopped ∼7 days after departing the Rhode Island coast. Interaction with a vessel is supported by the relatively constant ground speeds and altitudes observed on days 2 and 6 of Chip's offshore movements (Figure S3). For these reasons, we have included only the first day of Chip's offshore movements in our analysis.

Our spatio-temporal analyses of the trans-oceanic movements of 10 juvenile Ospreys show that these birds flew relatively constant course track segments (n = 25) that ranged between 105 and 1572 km in distance, 2.3 and 43.9 h in duration, 29 and 61 km/h in ground speed, and 160° and 230° in ground bearing (Table 1). Straightness index values for these track segments ranged between 0.9190 and 0.9996. The average distance travelled per track segment was 580.7 (±404.8) km, with average duration of 15.1(±10.8) hours, average ground speed of 44.9(±8.7) km/h, and average ground bearing of 192.9°(±18.0°). The average straightness index value was 0.9893(±0.0164).

### Compensation for Wind

Our wind analysis is different to a wind analysis of European osprey tracks [1] in three key ways. First, the size of our dataset is considerably larger. Our dataset includes 269 high resolution GPS locations from 10 different juvenile ospreys during 14 days of migration over the western Atlantic Ocean, whereas the earlier analysis is based on 23 lower resolution satellite transmitter locations from 2 different juvenile ospreys during 55 days of migratory movement over continental Europe and Africa [1]. Thus, the data we present is approximately 5 times the spatial and temporal resolution of the only other wind drift analysis of juvenile osprey movements. Second, our analysis is based on modelled meteorological conditions at the time and location of the tracked birds, whereas the earlier analysis was based on linearly interpolated reanalysis data with a 2.5° lat/long grid spacing at 12:00 UTC [1]. Third, we do not assume preferred migratory directions in this research. The assumption of preferred migratory direction is a fundamentally flawed approach for several reasons. Most importantly, such an approach assumes that direction is a key orientational cue in the navigational system being used by the animal. This may, or may not, be the case. We strongly believe such key facts must be independently established prior to assuming not only its existence, but also its quantitative value in a specified coordinate space. Assuming preferred migratory directions is also flawed in that it is extremely likely to pre-determine the findings of any wind vector analysis: individual differences in navigational behaviour are likely to be misinterpreted as the consequence of wind drift, rather than true navigation. Thus, rather than assume preferred migratory directions, we used the groundtrack directions actually flown by individual birds in our wind vector analysis. Groundtrack directions were determined using piecewise linear regression breakpoint analysis of the high-resolution GPS-enabled PTT location data (see *Statistical Analyses*, above).

Central to the justification of this approach to wind vector analysis is the recognition that birds flying through dynamic winds would not be expected to follow constant course, nor constant velocity ground tracks in the absence of drift compensation. All flying objects are sensitive to the winds they are moving through. As a consequence, dynamic winds must produce dynamic ground track directions and variable ground speeds in the absence of wind compensation. By combining the observed high-temporal resolution ground-track directions flown by juvenile ospreys with sophisticated atmospheric circulation models, we were able to determine each bird's individual response to wind at the hourly scale. This high-resolution approach to wind vector analysis improves on earlier coarse resolution studies as it allows us to more accurately identify individual movement behaviours in response to dynamic winds using objective quantitative methods.

The ten juvenile ospreys we tracked experienced dynamic winds during their trans-oceanic movements. Wind directions, determined for the time and location of each GPS transmission, spanned a 325° range with wind speeds ranging between 2 and 70 km/h. The mean wind direction was out of the northeast (55.5°±85°; ±1σ), and the mean wind speed was 27.3 km/h (±13.5 km/h; ±1σ).

**Figure 4:**
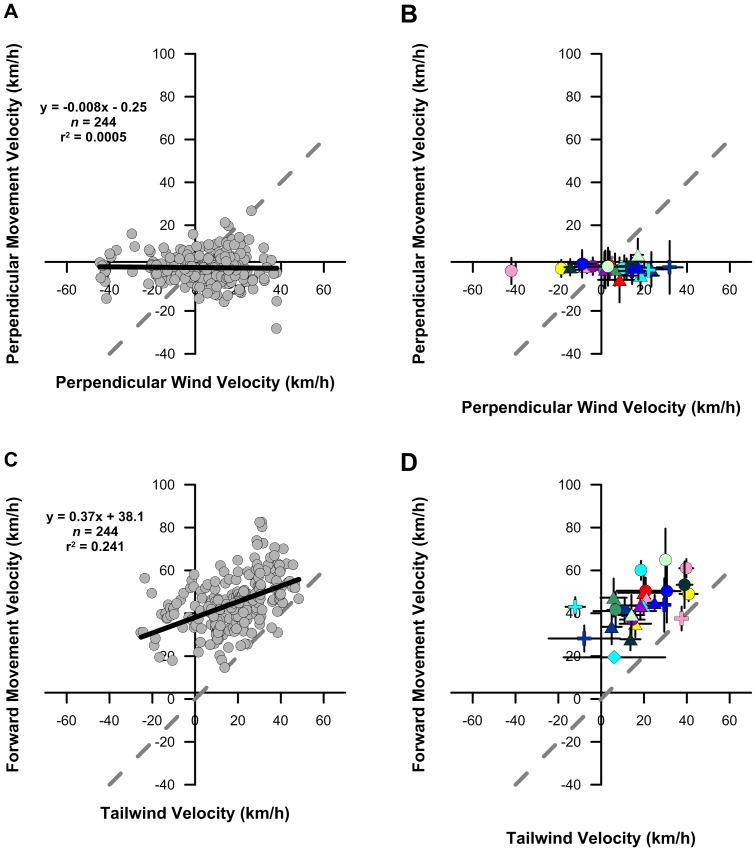
Wind vector analysis. Relationship between wind velocity and juvenile osprey movement velocity perpendicular to (A, B) and parallel to (C, D) the mean ground track direction of migration track segments. Wind velocities were determined for one-hourly GPS locations (A, C) using a dynamic regional mesoscale meteorological model. Track segment mean velocities (B, D) ±1σ error bars are also shown. Track segment colors and symbols as in Figure 1. Dashed lines represent 1:1 relationship.

In contrast to juvenile ospreys migrating over land [1], our results demonstrate that juvenile ospreys migrating over open ocean fully compensate for the effects of perpendicular wind drift (Figure 4A-B). Our analyses reveal that there is no significant correlation between the birds' perpendicular movement velocities and corresponding perpendicular wind velocities (p = 0.734; ANOVA; F = 0.12; df_reg_ = 1; df_err_ = 242; α = 0.05). Despite experiencing perpendicular winds as strong as 40 km/h, both from the left and from the right of their forward movement direction, the birds we tracked were rarely off course by more than 10 km (Figure 4A). Thus, we reject the hypothesis that juvenile ospreys experience full wind drift during long-distance migration over open ocean. Rather, the data we report demonstrate that not only did these birds compensate for wind drift, but they overcompensated (i.e. perpendicular movement and wind velocities with opposite signs) for the effects of perpendicular wind drift more than 51% of the time.

Our analyses also revealed that there is a significant positive linear covariation between forward movement velocity and tailwind velocity (p<0.05; ANOVA, F = 51.98; df_reg_ = 1; df_err_ = 242; α = 0.05; Figure 4C-D). This finding indicates that the birds' forward movement velocities were higher when they experienced stronger tailwinds, as would be expected for a bird experiencing tailwind support [40]. In fact, approximately 88% of the bird locations in our wind vector analysis returned positive tailwind velocities, demonstrating that the birds we studied experienced tailwind support during a large majority of their time aloft. These results are further supported by the similarity between the zero-wind forward movement velocity value we report (38.0 km/h) and the values reported by others [39], [41].

**Figure 5:**
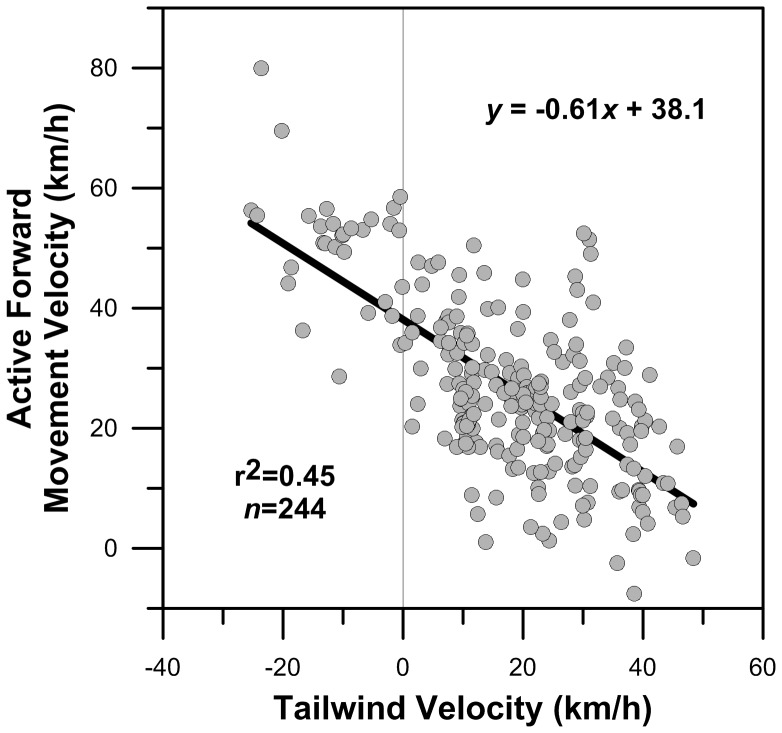
Relationship between osprey airspeed and tailwind velocity. Symbols correspond with one-hourly osprey GPS-enabled PTT tag locations. Linear least-squares regression equation is represented as the solid black line.

We were surprised to find a highly significant negative linear covariation between active forward movement velocity and tailwind velocity (p<<0.05; ANOVA, F = 189.08; df_reg_ = 1; df_err_ = 242; α = 0.05; Figure 5). This finding demonstrates that birds experiencing stronger tailwinds maintained lower active forward movement velocities, as would be expected given the energetic demands of non-stop long-distance migration. However, our results also show that birds experiencing headwinds (i.e. negative tailwinds) maintained the highest active forward movement velocities, often in excess of 50 km/h (Figure 5), rather than allowing their forward movement velocities to decrease. Such adjustments to forward movement velocity in response to variable winds is consistent with optimal bird migration theory, whereby the maintenance of flight speed minimizes either the overall energetic cost or flight duration of a bird's migration [42], [43]. However, the orientational and navigational requirements of migratory flight optimization are not insignificant. In order to optimize its movement behavior by increasing/decreasing flight speed in response to changing winds, a bird must first be able to determine that its forward movement velocity has changed: a process that requires not only a mechanism of positional orientation but also a mechanism for determining the passage of time.

Our wind analyses reveal two key findings relevant to the study and interpretation of avian migration. First, in contrast to juvenile ospreys flying over land, our wind analyses demonstrate that juvenile ospreys flying over open ocean have the ability to fully compensate for the effects of perpendicular wind drift. When combined with the extreme straightness of the track segments, these results demonstrate remarkable positional orientation (i.e. ‘map’ sense) despite the perturbing effects of variable wind speeds and wind directions. Second, our analyses further demonstrate that juvenile ospreys migrating over ocean compensate for headwind displacement. These findings indicate that juvenile ospreys not only adjust their heading direction, but also their airspeed, in order to maintain forward movement progress through time along constant course flight paths.

**Figure 6:**
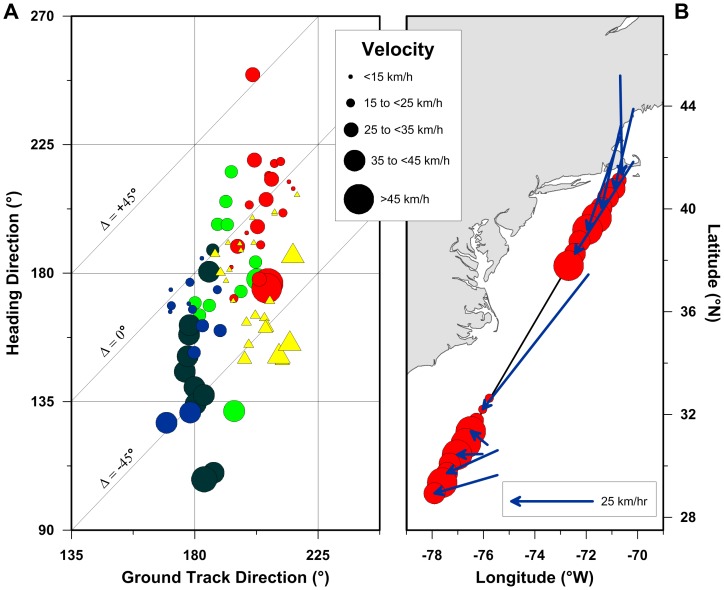
Hourly-scale juvenile osprey navigational responses to wind during trans-oceanic migration. Symbols and colors as in Figure 1. Symbol size is proportional to wind velocity (A) and forward movement velocity (B) as shown in the velocity legend. Blue vectors (B) correspond with the wind direction and wind speed (scale bar shown) experienced by the juvenile osprey ‘Felix’ during his>1500 km constant course movement between Martha's Vineyard and the Bahamas between the morning of September 16 and evening of September 17, 2007.

These two insights into juvenile osprey migratory behavior are best demonstrated by the hourly-scale PTT tag locations and associated WRF wind vector data for individual track segments. The five longest juvenile osprey track segments we observed, all in excess of 1000 km, spanned at least 18 hours of non-stop flying over open ocean (Table 1). Hourly-scale analysis of these data reveals that each of these track segments describe remarkably constant course paths despite the presence of highly variable wind speeds and directions across the track paths (Figure 6). In order to maintain the observed constant course groundtrack directions, these birds had to change their heading directions by as much as 90° (Figure 6A). This high-resolution analysis further demonstrates that when these birds experienced stronger winds, they followed heading directions that were as much 76° oblique to their near constant groundtrack directions, and followed heading directions that were similar to their groundtrack directions when winds were lighter (Figure 6A). It is worth noting that had these birds experienced full-wind drift, and not compensated for the effects of wind displacement, our high-resolution analysis would have shown the opposite pattern to what is presented in Figure 6A. In the case of full-wind drift, the data would plot as near constant heading directions with highly variable groundtrack directions due to the extremely dynamic nature of the winds these birds flew through.

The temporal compensation for the effects of wind displacement are also clearly demonstrated by hourly-scale analysis of the PTT tag and WRF wind data. For example, during the morning hours of the juvenile osprey Felix's second day aloft, the winds he flew through changed dramatically in both direction and speed (Figure 6B). Yet, not only did ‘Felix’ maintain his constant course groundtrack direction (Batschelet Straightness  =  0.9983; Table 1), he also maintained his near constant ∼50 km/h groundspeed by tripling his active forward movement velocity to more than 60 km/h following this shift in winds (Figure 6B). By combining high-resolution PTT tag with spatially and temporally matched WRF data, we can clearly see and quantify the compensatory movement behaviors performed by these birds during their constant course long distance movements.

Collectively, these results indicate that the navigational system utilised by juvenile ospreys during trans-oceanic migration enables spatially precise and temporally modulated positional orientation at the kilometer to sub-kilometer spatial scale over periods of less than one hour during both day and night. The fundamental question that emerges from these findings is: how do ospreys achieve such precise positional orientation?

### Mechanisms of Positional Orientation

It is widely accepted that drift compensation requires navigation. In conceptual terms, an animal correcting for displacement by currents must be able to determine whether or not it is off course, or out of position, in a defined coordinate space [44]. Yet, we still cannot explain in a mechanistic way the movement behaviors, such as wind drift compensation, animal tracking technology has enabled us to observe.

In an effort to explore how ospreys compensate for wind drift, we focus the following discussion on the juvenile osprey's sense of positional orientation. Several mechanisms of positional orientation [11] can be ruled out immediately given the spatial and temporal context of the movements we observed. First, visual cues, including topographic features and the position of celestial bodies, are unlikely candidates as the constant course movements we report are over open ocean and often occur across time periods in excess of 12 hours spanning both hours of daylight and darkness. Thus, there are effectively no available visible landmarks for beaconing, and any visible celestial bodies will have markedly changed position during a single constant course track segment, including the altitude of the celestial pole of rotation. Second, olfactory cues, although likely sources of homing information, are unlikely to carry the necessary positional information required for wind drift compensation over open ocean at the spatial and temporal scales observed. Olfactory cues must be as spatially dynamic as the winds that carry them, yet, our analyses reveal that variable winds have little effect on juvenile osprey navigation. Third, infrasonic cues are also unlikely as surf, the only relatively continuous source of infrasound, must be sourced from the coastline. Even if infrasonic cues from the North American coast were detected across hundreds of kilometers of ocean, it is difficult to imagine these cues providing the positional information required for the constant course, but variably subparallel to the coastline, movements these ospreys performed.

This leaves magnetic and gravitational cues [11] as two possible sources of positional orientation compatible with the movement behaviors we observed. These are attractive possibilities as they are both ubiquitous and relatively stable. However, we will not consider gravitational cues here as they require a sophisticated geophysical analysis of the Sun-Moon-Earth three-body problem [45] in addition to incorporation of gravitational anomalies associated with the structure and composition of earth's interior. We instead focus our discussion on magnetic cues. In contrast to the few studies that considered the possibility of gravitationally informed animal orientation [46]–[50], the idea that animals could navigate using bicoordinate positional information derived from the magnetic field is more than 130 years old [51].

### Bicoordinate Positional Orientation in Magnetic Space

Several different animal groups, including reptiles [44], birds [48], fish [52], mammals [53], insects [46], crustaceans [54], and amphibians [55] are believed to navigate using information derived from the magnetic field. Within these groups, several species such as sockeye salmon (*Onchorynchus nerka*) [56], loggerhead sea turtles (*Caretta caretta*) [21], spiny lobsters (*Panulinus interruptus*) [54], nightingales (*Luscinia megarhynchos*) [57], and red-spotted newts (*Notophthalmus viridescens*) [58] have been interpreted to use magnetic ‘maps’ for positional orientation, with the most widely hypothesized bicoordinate maps based on magnetic field intensity (F) and magnetic inclination (I).

**Figure 7:**
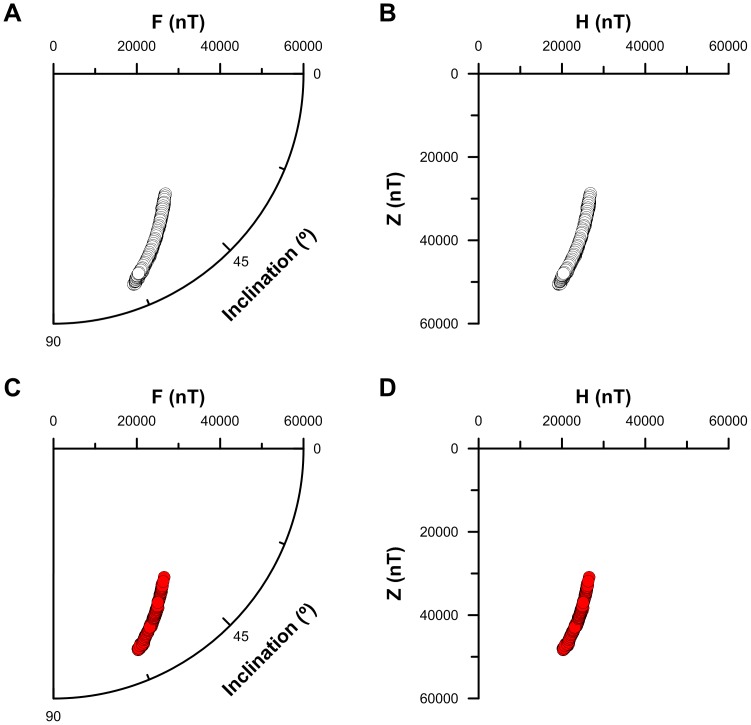
Vertical plane bicoordinate geomagnetic space in the Western Atlantic Ocean region. Polar (A, C) and Cartesian (B, D) plots of geomagnetic field coordinates for a 0.5° latitude-longitude grid between 20° and 42° north latitude and −67° and −78° west longitude (A, B; n = 1035) and all of the one-hourly juvenile osprey GPS-enabled PTT tag locations we report (C, D; n = 244 locations from ten birds). The {0;0} coordinate space origin in all panels corresponds with the subcrustal origin of Earth's main magnetic field.

However, it is important to understand that a magnetic F-I bicoordinate map is very much different than a common geographic bicoordinate map. The F-I map differs most from the physical maps humans are familiar with in that it is entirely conceptual if not cognitive and not a tangible object. Yet, even at the conceptual level it is quite different than traditional bicoordinate geographical maps. First, the F-I magnetic map is in a polar coordinate system and is not a Cartesian projection of two angular coordinates, like most latitude-longitude maps. Thus, unlike latitude and longitude, F and I are not orthogonal coordinates by definition. Second, as must be the case in a polar coordinate system, F and I have different units: one is an angle (I) and one is a scalar quantity (F). Thus, the F-I map is more like an altitudinal (i.e. F) cross-section spanning a latitudinal (i.e. I) range than it is like a true geographic map. This important distinction is reinforced by the equivalence of the polar F-I coordinate system to the orthogonal Cartesian magnetic vertical intensity (Z) versus magnetic horizontal intensity (H) coordinate space (Figure 7). Third, F and I are not uniformly distributed due to the irregular and unpredictable nature of Earth's magnetic field. For example, there is a much higher probability of randomly selecting a location with an inclination of 65° than there is of selecting a location with an inclination value of 25° [59]. Fourth, and most relevant to the topic of animal navigation, F and I are strongly autocorrelated across large areas of Earth's surface due to the predominantly dipolar nature of the magnetic field [60], [61]. This autocorrelation exists because steeper inclination angles and higher field intensities are found closer to the magnetic poles, while shallower inclination angles and lower field intensities are found closer to the magnetic equator. As a direct consequence, not all bicoordinate combinations of F and I values are possible: random animal movements and highly orientated movements are likely to be indistinguishable in F-I bicoordinate space as it is effectively one dimensional across large portions of Earth's surface. This geometric paradox is best represented by a simple analogy: even the most skilled navigators' plots appear no different than random walks when their maps are viewed from the side.

Yet, the question remains valid: is bicoordinate F-I positional orientation compatible with the trans-oceanic movement behaviors of juvenile ospreys? To answer this question, we first had to determine what model best describes the F-I coordinate space the birds moved through. This was accomplished using Enhanced Magnetic Model coordinate locations (n = 1035), determined for a latitude-longitude coordinate grid with 0.5° spacing across the geographic spatial domain of the bird movements (i.e. 20°N to 42°N; −67°W to −78°W). A 4^th^-order polynomial model (r^2^ = 0.99614), fit to a Cartesian H-Z representation of the polar F-I data, was significantly different than a 3^rd^-order polynomial (r^2^ = 0.99611), but not a 5^th^-order polynomial (r^2^ = 0.99614). We used this 4^th^-order polynomial model to test the hypothesis: juvenile osprey migration track segment F-I coordinate trajectories are not significantly different. We performed this test by: 1) fitting a 4^th^-order polynomial to each of the migration track segments reported in Table 1; 2) performing a multiple ANOVA (t-test) on all possible track segment pairs; 3) identifying which correlation coefficients for which track pairs were significantly different (α = 0.05).

Surprisingly, we found that none of the 4 regression coefficients for any of the 300 paired track segments were significantly different (Table S1; Figure 7). We therefore conclude that there is no significant difference between any of the juvenile osprey movements we report in F-I coordinate space. The most likely explanation for this result is that autocorrelation between F and I values in the western Atlantic cause all locations across the region to fit the same 4^th^-order polynomial relationship (Figure 7). These findings demonstrate that the juvenile ospreys movements we report are not compatible with navigation in bicoordinate geomagnetic F-I space.

Our wind vector analyses further support this interpretation. Integration of our wind vector analysis and our geomagnetic coordinate analysis reveals that even if these birds had been navigating in F-I space, they would not have been able to compensate for their displacement by wind due to the autocorrelation between F and I values across the region. In other words, there is insufficient spatial resolution in F-I magnetic coordinate space across the western Atlantic to explain the wind drift compensation these birds perform. Thus, the constant course movements we report are not compatible with the bicoordinate F-I ‘magnetic map’ system of positional orientation that is being utilized by other species inhabiting the same region [21], [54]. These conflicting interpretations reinforce a paradox others have raised before: how can a species navigate through a coordinate space with very limited spatial resolution [60], [61]? One possibility is that not only do different species navigate in different ways, but that they also have very different sensitivities to magnetic field conditions. Yet, there is at least one alternative: perhaps we’re looking at the magnetic coordinates from the wrong perspective.

### Transformed Magnetic Coordinate Maps

The decision to define magnetic coordinates relative to a subcrustal origin is arbitrary, particularly from a migrating animal's perspective. We argue that the magnetic cues many animals show orientational responses to are more likely to be defined relative to a location that is meaningful to the animal, such as the location of home. Transforming the origin of a coordinate space involves a series of simple matrix operations, and the juvenile ospreys we tracked are ideally suited to this type of magnetic coordinate transformation as we know both the natal location and year of hatching for each bird. Thus, we can transform the data to a nesting site origin, and we can reasonably ignore the temporal complexities of magnetic secular variation and questions regarding magnetic remanence given the <1 year-old age of each bird. By transforming the magnetic data, we are effectively changing the perspective from which the coordinate space is being viewed: we are no longer looking at our navigators' maps edge on, but from an entirely new perspective.

We rotated, tilted, and translated the Enhanced Magnetic Model {X, Y, Z} Cartesian coordinate data determined for our juvenile osprey migration tracks by the magnetic declination, inclination, and field intensity of each bird's nesting site on May 1 of the year hatched. This coordinate transformation yields the new Cartesian magnetic coordinates {*x_T_; y_T_; z_T_}*, with *x_T_* representing an axis parallel to the magnetic field lines at the nest, *y_T_* representing an axis perpendicular to the magnetic field lines at the nest in the horizontal plane, and *z_T_* representing an axis perpendicular to the magnetic field lines at the nest in the vertical plane. Each of these axes is expressed in the same units (Tesla or Gauss), as the Cartesian geomagnetic coordinates {X, Y, Z}. The key difference is that in transformed magnetic coordinate space a {*x_T_; y_T_; z_T_}* location of {0, 0, 0} is equivalent to the nesting site location, rather than the >5000 km deep origin of Earth's geomagnetic field.

If we consider the magnetic field lines at the nest as representing a magnetic pole, then the *y_T_-z_T_* plane is the plane perpendicular to this magnetic pole. Thus, any movement by the bird away from the nesting site (i.e. away from its transformed magnetic pole) will result in a change in the bird's location in *y_T_-z_T_* bicoordinate space as the orientation of the magnetic field lines change along the bird's path. As a complete analysis of the juvenile osprey movements in a three-dimensional transformed magnetic coordinate space is beyond the scope of this study, we restricted our interpretation of the observed trans-oceanic juvenile osprey movements to the *y_T_-z_T_* coordinate plane. We chose to focus on this bicoordinate system for the following reasons: 1) it is an orthogonal Cartesian coordinate space rather than a polar coordinate space; 2) any change in magnetic inclination angle, the parameter most widely associated with the biological magnetic sense, will change the *z_T_* coordinate value but not the *y_T_* coordinate value; 3) movements away from the nesting site along different geographic bearings will appear as different *y_T_-z_T_* trajectories due to the sensitivity of these two coordinates to both magnetic declination and inclination.

**Figure 8:**
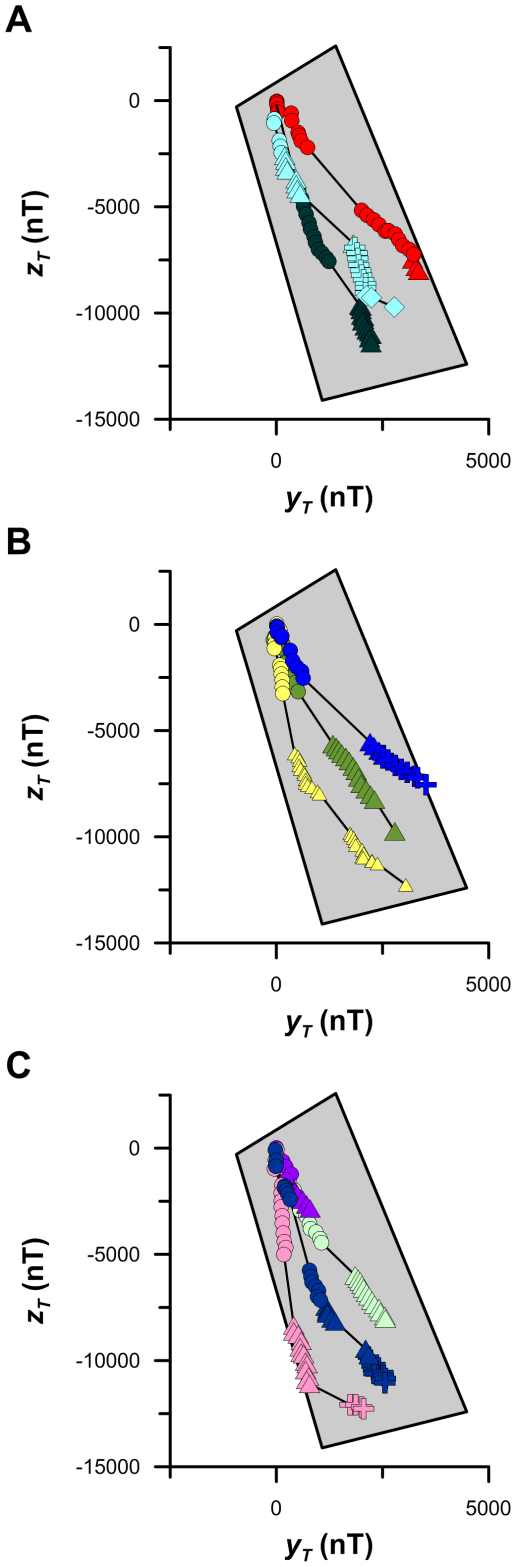
Transformed magnetic coordinate osprey locations. Colours and symbols as in Figure 1. Gray shaded area corresponds with the area encompassed between 20° and 42° north latitude and −67° and −78° west longitude. The origin of the transformed magnetic coordinate space shown here corresponds with each bird's hatching site (see text).

Transformed magnetic coordinate maps (*y_T_-z_T_* plane) of the juvenile osprey movements show several notable results. Most importantly to the topic of navigation, the different tracks follow different trajectories in *y_T_-z_T_* space (Figure 8). This is distinctly different than what was shown above for the same track data expressed in F-I bicoordinate space. Multiple ANOVA (t-test) performed on 300 different pairs of the 25 track segments we identified show that >81% of these pairs are significantly different in transformed magnetic coordinate space (Table S2). Thus, unlike the F-I coordinate space, there is considerable resolution (ca. ∼6,000 nT × ∼17,000 nT) in the *y_T_-z_T_* plane across the trans-oceanic migratory spatial domain spanned by these birds. The observation that most of the track segments follow different transformed magnetic trajectories is also consistent with the fact that we know these birds followed different wind-drift compensated constant course track segments in geographic space. The results of a similar multiple ANOVA performed on the same track segment pairs, shows that >77% of the track segment pairs are significantly different in Mercator coordinate space (Table S3).

In addition to being spatially distinct, the osprey track segments are also very straight in *y_T_-z_T_* space (Table 2). Straightness index values in *y_T_-z_T_* space range between 0.7772 and 1.0000. Of the twenty-five track segments identified, twenty-two have *y_T_-z_T_* straightness index values>0.9500 and nine have higher straightness index values in *y_T_-z_T_* space than they do in Mercator space. Interestingly, the Mercator and *y_T_-z_T_* straightness index values we determined are not significantly different (*t*-test; df = 48; *t* = 1.68; p = 0.11; Table 1, Table 2). Although the migratory movements of the juvenile ospreys we tracked are straighter in Mercator coordinate space (mean straightness index  =  0.9893) than in *y_T_-z_T_* space (mean straightness index  =  0.9731), we cannot determine with confidence which of these two coordinate systems better represents the navigational coordinate space of juvenile ospreys.

**Table 2:** Track segment straightness in transformed magnetic coordinates.

Osprey (segment)	Straightness Index (*y_T_* vs. *z_T_*)	*z_T_* Standard Error (nT)	*y_T_* Standard Error (nT)
Bea (1)	0.9516	350.0	63.5
Bea (2)	0.9965	107.6	37.3
Belle (1)	0.9959	424.7	18.3
Belle (2)	0.9872	454.6	49.5
Belle (3)	0.9834	39.3	74.6
Caley (1)	0.9994	104.5	14.1
Caley (2)	0.9826	336.2	137.1
Caley (3)	0.9962	144.2	18.7
Caley (4)	0.9999	4.3	5.1
Felix (1)	0.9616	244.4	110.1
Felix (2)	1.0000	4.5	1.2
Henrietta (1)	0.9790	229.5	56.8
Henrietta (2)	0.9913	55.7	19.6
Isabel (1)	0.9947	205.5	31.4
Isabel (2)	0.9861	120.7	57.9
Isabel (3)	0.7772	186.0	75.6
Luke (1)	0.9951	220.7	45.1
Luke (2)	0.9699	176.6	29.9
Mittark (1)	0.9402	115.7	30.5
Mittark (2)	0.9998	7.9	4.0
Mittark (3)	0.9938	34.2	28.2
Moffet (1)	0.9898	719.0	48.1
Moffet (2)	0.9659	287.3	111.4
Chip (1)	0.9052	234.3	73.6
Chip (2)	0.9864	47.7	20.8
**Average (±1σ)**	**0.9731 (±0.0465)**	**194.2 (±168.6)**	**46.5 (±35.4)**

Determining the coordinate space of animal orientation during long-distance migration is a crucial step in understanding how animals navigate. It is also relevant to the animal tracking community as widely applied state-space models assume that animal behaviors can be inferred from changes in an animal's movements in geographic coordinate space [62]. In fact, many published migration tracks are often interpolated in geographic coordinates from a limited number of data points using state-space modelling methods [63]. It is important to realize that the results produced by geographic coordinate system state-space models may be inaccurate if the animal studied was actually navigating in magnetic (e.g. F-I) or transformed magnetic (e.g. *y_T_-z_T_*) coordinate space due to the irregular and temporally dynamic shape of the magnetic field at the Earth's surface. The effects of analysing animal track data in one coordinate space versus another is an important but understudied problem in animal tracking research and we encourage future researchers to explore this issue in some detail.

The transformed magnetic coordinate system we analysed represents a promising new perspective on magnetic map navigation during long-distance migration. It both overcomes many of the challenges that have been identified for magnetic F-I bicoordinate maps (i.e. autocorrelation), and is also compatible with the experimental results that provide the empirical basis for the F-I map hypothesis [64]. First, any behavioral response to a change in magnetic inclination must also be considered as a response to a change in *z_T_* as the two parameters are geometrically related: all else being equal, a change in one of these parameters must be accompanied by a change in the other. Thus, the transformed coordinate space we present supports the large number of experimental studies that have shown orientational responses to changes in magnetic inclination. Second, the *y_T_-z_T_* coordinate space is conceptually more like a map than the F-I bicoordinate space. For example, in contrast to the polar F-I space, the orthogonal geometry of *y_T_-z_T_* space facilitates orientational and statistical analyses that are familiar and simple (e.g. determination of *y_T_-z_T_* directional bearings; linear regression versus 4^th^-order polynomial regression). Third, the *y_T_-z_T_* coordinate space does not suffer from autocorrelation between the two coordinate parameters as the *y_T_-z_T_* space is sensitive to changes in both magnetic declination and inclination. Fourth, the *y_T_-z_T_* coordinate space origin is defined relative to a biologically meaningful location: the location of the animal's origin. As many migratory animals exhibit high levels of site fidelity, it seems plausible that their movements will be made in a coordinate space that is relative to the natal areas that have a proven capacity of supporting animal fitness and survival. As we were unable to identify a significant difference between trans-oceanic juvenile osprey movements in Mercator and transformed magnetic coordinate spaces, future research aimed at testing for significant differences between these different spatial representations of animal movement data is a priority.

### Chord and Clock Navigation

Our analysis of the trans-oceanic portion of juvenile osprey migration track data has revealed several new insights into the navigational behaviors and capacities of these birds. The observation that naïve ospreys are capable of maintaining remarkably precise constant course movements despite the effects of variable winds demonstrates they have a profound ability to locate themselves in at least one coordinate space. The observation that these same birds are compensating for the effects of headwinds further demonstrates that their movements are also paced through some means of keeping time. Collectively, these two observations suggest that the juvenile osprey navigational system integrates both positional and temporal information.

The only theoretical framework of animal orientation that explicitly requires temporal information is vector navigation (i.e. endogenous spatio-temporal programming). However, vector navigation does not involve positional orientation and is therefore incapable of explaining an animal's ability to compensate for displacement by currents. Thus, none of the existing theoretical frameworks of animal navigation are capable of explaining the movement behaviors of juvenile ospreys migrating over open ocean. Rather, a navigational system that includes both positional and temporal information is required.

For example, if we assume the ospreys we tracked have an ability to monitor their bicoordinate spatial position through time, the effects of wind drift may be transduced and compensated for by responding to the displacement velocity relative to some datum. This concept is similar to the inertial navigation systems that have been widely used in ships, aircraft, and submarines for decades, and was first proposed for the purpose of animal navigation at least 50 years ago [65]. Yet, the system we propose is fundamentally different from inertial navigation in that the possible sources of both positional and temporal information are external to the animal (e.g. magnetic field; celestial cues). Thus, the two fundamental components of the animal navigation system we propose are the *chord*, here defined as the scalar distance or gradient between two locations in a specified coordinate space, and the *clock*, here defined as a natural mechanism for gauging the passage of time that is calibrated against exogenous time dependent cues.

The *chord and clock* system has a distinct advantage over the widely accepted *map and compass* system of navigation: it provides a means of solving an animal's core ecological need of arriving in a biologically suitable habitat at a biologically suitable time. For example, a humpback whale would not be well served by its skills of precision navigation if it were to arrive on its feeding grounds well after, or well before, the annual bloom in primary productivity. Similarly, animals that mate during or shortly after seasonal migrations need to find their mates in a time period that is conducive to reproductive success: arriving in the right place at the wrong time does little good for these animals. One of the best examples of the integration of spatial and temporal information during animal movement are the annual migrations of Christmas Island red crabs (*Gecarcoidea natalis*).

There is excellent evidence that Christmas Island red crab movement behaviors are temporally modulated by environmental cues [66]. Observation of the 1993 and 1995 migratory movements of Christmas Island red crabs showed that females spawned in the pre-dawn hours of Dec. 12 and 13 in 1993 and Dec. 17 in 1995, as predicted by previously observed reproductive synchronicity with the tidal gravity cycle [67]. Radio-tracking also revealed that crabs followed similar constant course movements toward the same coastal area in both years, despite the possibility of reaching a different section of coast located half as far away but in the opposite direction. Surprisingly, the onset of crab migration from inland forests to coastal environments was 3 weeks earlier in 1995 than in 1993, presumably due to the late arrival of seasonal rain in 1993. During the 1993 Christmas Island red crab migration, the mean daily rate of movement was more than double the mean daily rate of movement in 1995 [66]. Like the juvenile osprey data we report here, these red crabs demonstrated a remarkable temporal pacing of spatially precise migratory movements.

Just as it is impossible to compensate for the effects of wind drift using only directional orientation, it is similarly impossible to compensate for temporal shifts caused by environmental conditions, such as the late arrival of seasonal rain, based solely on endogenous biological rhythms. Rather, the movement behaviors of ospreys and red crabs at the very least require a mechanism for calibrating their clocks relative to some exogenous cue following environmental disturbances.

In the case of the red crabs, the data suggest that the tidal gravity cycle provided exogenous time dependent information that elicited a response in the crabs' migratory movement behavior. There are many other examples of animal movements that are synchronized to tidal gravity or lunar phase cycles, including: mate searching in crabs [68]; salt water entry by salmon smolts [69]; petrel flight schedules [70]; and shark, tuna and seal diving [71]–[73]. However, the extent to which there is a causal link between these behaviors and their luni-solar correlates, and whether or not such tidal pacing is apparent in juvenile osprey movements, remains an open question.

Future experimental and field based research into the possibility of an integrated spatial and temporal system of navigation, like the chord and clock system we propose, is required to fully explore what these relationships can, and cannot, tell us about the grand challenges of organismal biology. We encourage further interdisciplinary work targeting: 1) ultra-high resolution (i.e. minute and meter scale) analysis of animal movements in comparable resolution wind/current fields in an effort to better capture the dynamics of animal navigation at the spatial and temporal scale at which navigational decisions are made; 2) analysis of animal movements in all possible magnetic and geomagnetic bi/tri-coordinate spaces relative to diverse geophysical reference frames/data; 3) experimental testing of animal movement responses to changes in transformed magnetic coordinate conditions. Like many in the community, we strongly believe that it is only through the integration of diverse tools and multiple research perspectives that the complex problem of animal navigation can be solved.

## Conclusions

How do juvenile ospreys navigate during trans-oceanic migration? Although a complete answer to this question remains elusive, our research has provided several relevant findings. The demonstration that juvenile ospreys are capable of maintaining constant course movements across large expanses of open ocean, despite the perturbing effects of highly variable winds, demonstrates that these birds have a remarkable ability to locate themselves in space and gauge their rate of forward progress. We further demonstrate that juvenile osprey movement behaviors are not compatible with the bicoordinate geomagnetic field intensity-inclination orientation hypothesis due to the lack of spatial resolution in this representation of magnetic coordinate space in the western Atlantic Ocean basin. However, vertical plane and horizontal plane magnetic cues, defined in a transformed magnetic coordinate space relative to each bird's hatching site, provides sufficient spatial resolution for the highly individual movements we observe and is compatible with the observations of others that many animals respond to vertical plane magnetic field conditions.

By integrating high resolution animal tracking technology with sophisticated meteorological and magnetic models, we show that the trans-oceanic migrations of juvenile ospreys are best explained by a spatio-temporal system of navigation. The chord and clock navigational system we propose provides a new theoretical framework for the analysis and interpretation of animal movements in dynamic environments. This new perspective on one of biology's oldest questions provides an interdisciplinary framework for future research targeting the means by which animals navigate. There is growing consensus that solving the grand challenges of organismal biology requires integrated and interdisciplinary research approaches [16], [29], [34]. We hope that the integrated approach we apply here inspires further synergistic research involving scientists from a range of disciplines.

## Supporting Information

Figure S1 **Platform transmitter terminal GPS and argos-doppler locations of adult and juvenile ospreys tracked in northeast North America between 2007 and 2012.** Red symbols correspond with juvenile osprey locations not included in the current study (68,623 locations for 26 individual ospreys), blue symbols correspond with adult osprey locations not included in the current study (7600 locations for 15 individual ospreys), and white symbols correspond with the ten juvenile ospreys we studied. We studied these ten birds due to the trans-oceanic nature of the initial phase of their southward migrations. Northing and Easting values are shown in kilometers.
(TIF)

Figure S2 **Platform transmitter terminal GPS track map of pre-migration movements for the ten juvenile ospreys studied.** Colours as in Figure 1 (pink  =  Belle; red  =  Felix; yellow  =  Moffet; light green  =  Henrietta; green  =  Bea; dark green  =  Luke; light blue  =  Caley; royal blue  =  Mittark; dark blue  =  Isabel; purple  =  Chip). Belle's southward return movement to Martha's Vineyard island was non-stop from her northernmost roost, and represents the longest continuous pre-migration movement performed by the ten juvenile ospreys we studied. Northing and Easting values are shown in kilometers.
(TIF)

Figure S3 **Flight altitude and ground speed versus time plot for the juvenile osprey ‘Chip’.** Chip's flight altitudes (A) and ground speed velocities (B) through time suggest that his anomalous, and likely fatal, eastward movement into the north Atlantic Ocean was the result of interaction with an oceanic vessel. The relatively constant velocity and low flight altitudes, particularly on days 2, 5 and 6, following departure from the coast, are consistent with the velocities and altitudes expected for a large vessel. Thus, we only included the first 24 hours of Chip's movements in our analysis.
(TIFF)

Table S1 **Probabilities that paired juvenile osprey migration segments are significantly different in geomagnetic bicoordinate space.**
(PDF)

Table S2 **Probabilities that paired juvenile osprey migration segments are significantly different in transformed magnetic bicoordinate space.**
(PDF)

Table S3 **Probabilities that paired juvenile osprey migration segments are significantly different in Mercator projection geographic bicoordinate space.**
(PDF)

File S1 **This file includes supporting text explaining how to use the freely downloadable magnetic models applied in this study.**
(DOCX)
