# Supplementary figures and images for: Juvenile Osprey Navigation during Trans-Oceanic Migration
Source: PLoS One. 2014 Dec 10;9(12):e114557. doi: 10.1371/journal.pone.0114557 (PMC4262435; doi:10.1371/journal.pone.0114557)

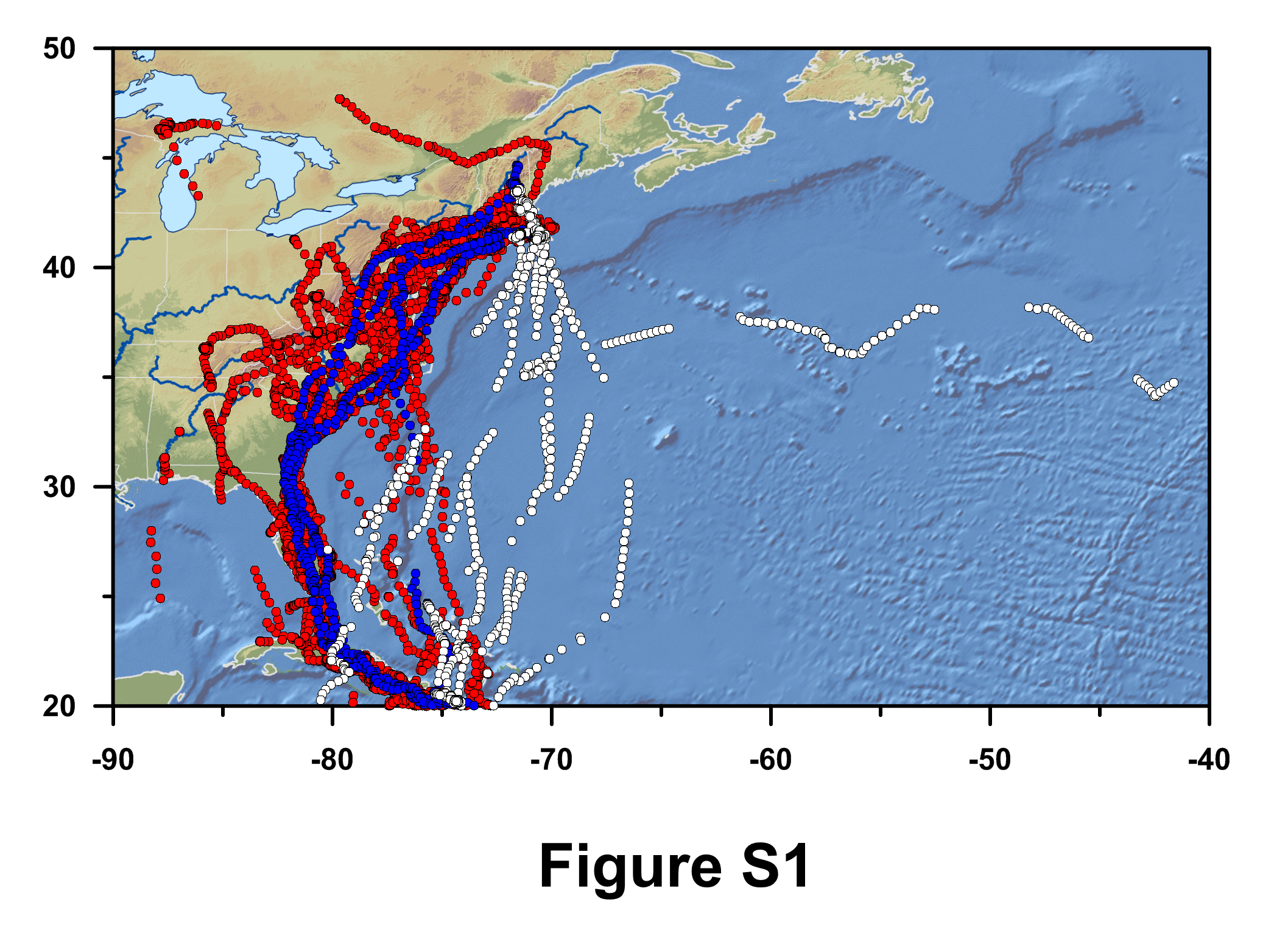

Supplement: Figure S1 — Platform transmitter terminal GPS and argos-doppler locations of adult and juvenile ospreys tracked in northeast North America between 2007 and 2012. Red symbols correspond with juvenile osprey locations not included in the current study (68,623 locations for 26 individual ospreys), blue symbols correspond with adult osprey locations not included in the current study (7600 locations for 15 individual ospreys), and white symbols correspond with the ten juvenile ospreys we studied. We studied these ten birds due to the trans-oceanic nature of the initial phase of their southward migrations. Northing and Easting values are shown in kilometers. (TIF) [file pone.0114557.s001.tif]

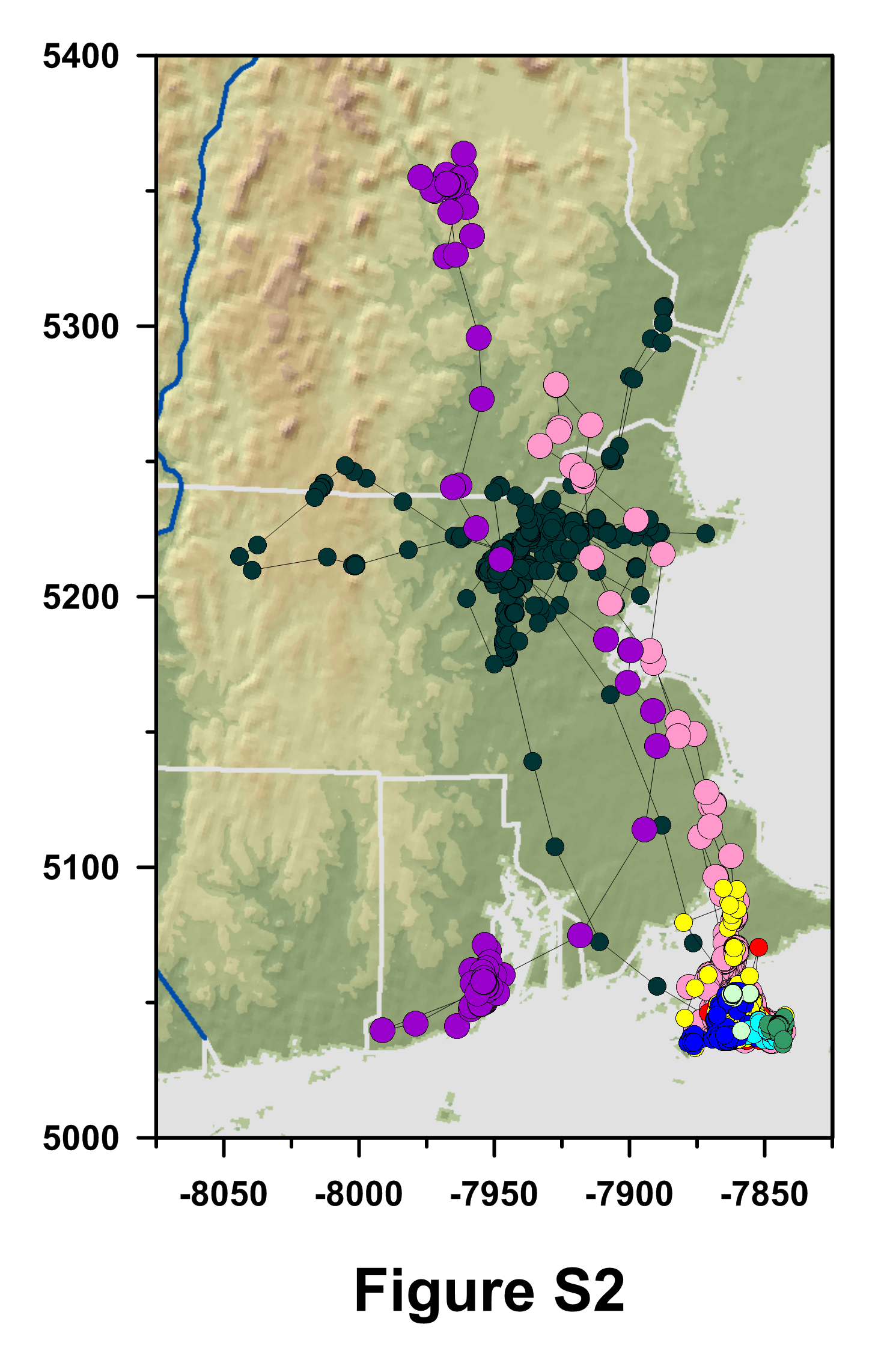

Supplement: Figure S2 — Platform transmitter terminal GPS track map of pre-migration movements for the ten juvenile ospreys studied. Colours as in Figure 1 (pink = Belle; red = Felix; yellow = Moffet; light green = Henrietta; green = Bea; dark green = Luke; light blue = Caley; royal blue = Mittark; dark blue = Isabel; purple = Chip). Belle's southward return movement to Martha's Vineyard island was non-stop from her northernmost roost, and represents the longest continuous pre-migration movement performed by the ten juvenile ospreys we studied. Northing and Easting values are shown in kilometers. (TIF) [file pone.0114557.s002.tif]

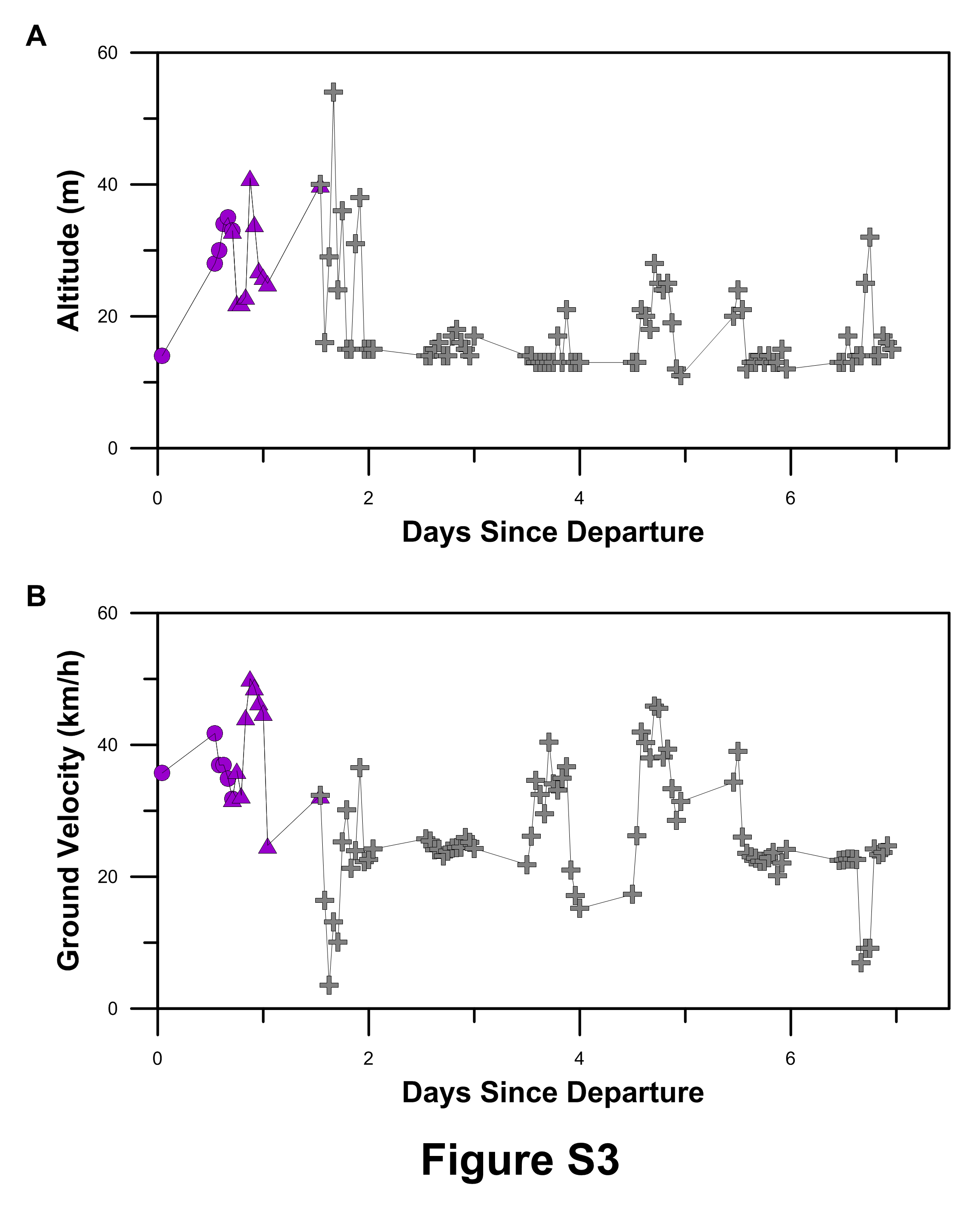

Supplement: Figure S3 — Flight altitude and ground speed versus time plot for the juvenile osprey ‘Chip’. Chip's flight altitudes (A) and ground speed velocities (B) through time suggest that his anomalous, and likely fatal, eastward movement into the north Atlantic Ocean was the result of interaction with an oceanic vessel. The relatively constant velocity and low flight altitudes, particularly on days 2, 5 and 6, following departure from the coast, are consistent with the velocities and altitudes expected for a large vessel. Thus, we only included the first 24 hours of Chip's movements in our analysis. (TIFF) [file pone.0114557.s003.tiff]
