# Supplementary material for: Juvenile Osprey Navigation during Trans-Oceanic Migration
Source: PLoS One. 2014 Dec 10;9(12):e114557. doi: 10.1371/journal.pone.0114557 (PMC4262435; doi:10.1371/journal.pone.0114557)
Supplement: Table S1 — Probabilities that paired juvenile osprey migration segments are significantly different in geomagnetic bicoordinate space. (PDF) [file pone.0114557.s004.pdf]

Table S1. Probability matrix of the comparison between 4th-order polynomial regression coefficients for paired osprey track segments in bicoordinate geomagnetic space.

	Bea (2)	Belle (1)	Belle (2)	Caley (1)	Caley (2)	Caley (3)	Felix (1)	Henrietta (1)	Henrietta (2)	Isabel (1)	Isabel (2)	Isabel (3)	Luke (1)	Luke (2)	Mittark (1)	Mittark (3)	Moffet (1)	Moffet (2)	Chip (1)	Chip (2)
**Bea (1)**	0.06 / 0.05 / 0.05 / 0.05	0.19 / 0.18 / 0.18 / 0.18	0.41 / 0.36 / 0.31 / 0.26	0.08 / 0.07 / 0.07 / 0.07	0.96 / 0.88 / 0.81 / 0.74	0.72 / 0.69 / 0.66 / 0.62	0.07 / 0.07 / 0.07 / 0.07	0.09 / 0.09 / 0.09 / 0.09	0.67 / 0.64 / 0.61 / 0.57	0.08 / 0.08 / 0.08 / 0.08	0.91 / 0.93 / 0.95 / 0.98	0.97 / 0.97 / 0.97 / 0.97	0.24 / 0.21 / 0.19 / 0.17	0.88 / 0.88 / 0.87 / 0.87	0.85 / 0.84 / 0.83 / 0.82	0.57 / 0.56 / 0.55 / 0.53	0.74 / 0.74 / 0.74 / 0.73	0.08 / 0.07 / 0.07 / 0.07	0.52 / 0.52 / 0.53 / 0.53	0.26 / 0.26 / 0.26 / 0.26
**Bea (2)**		0.23 / 0.22 / 0.21 / 0.20	0.88 / 0.88 / 0.87 / 0.87	0.68 / 0.66 / 0.63 / 0.59	0.05 / 0.05 / 0.05 / 0.05	0.91 / 0.90 / 0.89 / 0.88	0.36 / 0.36 / 0.35 / 0.35	0.34 / 0.34 / 0.33 / 0.33	0.98 / 0.98 / 0.98 / 0.98	0.35 / 0.35 / 0.35 / 0.34	0.75 / 0.75 / 0.75 / 0.74	0.98 / 0.98 / 0.98 / 0.98	0.50 / 0.51 / 0.51 / 0.51	0.90 / 0.90 / 0.90 / 0.90	0.08 / 0.08 / 0.08 / 0.08	0.70 / 0.71 / 0.71 / 0.72	0.07 / 0.07 / 0.06 / 0.06	0.41 / 0.40 / 0.40 / 0.39	0.63 / 0.63 / 0.63 / 0.63	0.28 / 0.28 / 0.28 / 0.28
**Belle (1)**			0.73 / 0.72 / 0.70 / 0.68	0.28 / 0.27 / 0.25 / 0.24	0.17 / 0.18 / 0.18 / 0.20	0.91 / 0.91 / 0.91 / 0.90	0.32 / 0.32 / 0.32 / 0.32	0.49 / 0.49 / 0.49 / 0.49	0.83 / 0.82 / 0.81 / 0.80	0.37 / 0.36 / 0.36 / 0.36	0.82 / 0.82 / 0.83 / 0.83	0.97 / 0.97 / 0.97 / 0.97	0.78 / 0.76 / 0.74 / 0.72	0.89 / 0.89 / 0.89 / 0.89	0.23 / 0.23 / 0.22 / 0.22	0.64 / 0.64 / 0.64 / 0.64	0.24 / 0.24 / 0.24 / 0.23	0.41 / 0.39 / 0.37 / 0.35	0.58 / 0.58 / 0.59 / 0.59	0.27 / 0.27 / 0.27 / 0.27
**Belle (2)**				0.97 / 0.97 / 0.96 / 0.95	0.41 / 0.39 / 0.36 / 0.33	0.97 / 0.96 / 0.95 / 0.94	0.85 / 0.85 / 0.84 / 0.84	0.83 / 0.82 / 0.82 / 0.81	0.94 / 0.94 / 0.93 / 0.93	0.84 / 0.84 / 0.84 / 0.83	0.77 / 0.77 / 0.77 / 0.77	0.98 / 0.98 / 0.98 / 0.98	0.83 / 0.83 / 0.83 / 0.83	0.89 / 0.90 / 0.90 / 0.90	0.37 / 0.33 / 0.29 / 0.25	0.69 / 0.69 / 0.70 / 0.70	0.48 / 0.44 / 0.39 / 0.34	0.86 / 0.86 / 0.86 / 0.86	0.62 / 0.62 / 0.62 / 0.62	0.28 / 0.28 / 0.28 / 0.28
**Caley (1)**					0.08 / 0.08 / 0.08 / 0.07	0.98 / 0.97 / 0.96 / 0.96	0.49 / 0.49 / 0.48 / 0.48	0.46 / 0.46 / 0.45 / 0.45	0.93 / 0.92 / 0.92 / 0.91	0.48 / 0.47 / 0.46 / 0.45	0.78 / 0.78 / 0.79 / 0.79	0.98 / 0.98 / 0.98 / 0.98	0.69 / 0.70 / 0.72 / 0.74	0.89 / 0.90 / 0.90 / 0.90	0.13 / 0.13 / 0.13 / 0.13	0.69 / 0.70 / 0.70 / 0.70	0.09 / 0.09 / 0.08 / 0.08	0.59 / 0.58 / 0.59 / 0.59	0.65 / 0.65 / 0.65 / 0.65	0.32 / 0.32 / 0.32 / 0.32
**Caley (2)**						0.73 / 0.72 / 0.70 / 0.69	0.05 / 0.05 / 0.05 / 0.05	0.07 / 0.07 / 0.08 / 0.08	0.68 / 0.67 / 0.65 / 0.63	0.06 / 0.06 / 0.06 / 0.05	0.90 / 0.91 / 0.93 / 0.94	0.97 / 0.97 / 0.97 / 0.97	0.23 / 0.22 / 0.21 / 0.20	0.88 / 0.88 / 0.88 / 0.88	0.81 / 0.75 / 0.68 / 0.63	0.58 / 0.57 / 0.57 / 0.57	0.76 / 0.83 / 0.90 / 0.97	0.06 / 0.05 / 0.05 / 0.05	0.53 / 0.54 / 0.55 / 0.56	0.28 / 0.28 / 0.28 / 0.28
**Caley (3)**							0.96 / 0.97 / 0.98 / 0.98	0.95 / 0.96 / 0.96 / 0.97	0.93 / 0.92 / 0.91 / 0.91	0.96 / 0.96 / 0.97 / 0.98	0.80 / 0.81 / 0.81 / 0.81	0.98 / 0.98 / 0.97 / 0.97	0.95 / 0.96 / 0.96 / 0.97	0.89 / 0.89 / 0.89 / 0.89	0.69 / 0.65 / 0.61 / 0.57	0.70 / 0.70 / 0.70 / 0.70	0.77 / 0.75 / 0.72 / 0.69	0.97 / 0.97 / 0.98 / 0.99	0.63 / 0.63 / 0.62 / 0.62	0.29 / 0.29 / 0.28 / 0.28
**Felix (1)**								0.78 / 0.77 / 0.76 / 0.75	0.88 / 0.87 / 0.87 / 0.87	0.81 / 0.81 / 0.82 / 0.83	0.80 / 0.80 / 0.80 / 0.80	0.97 / 0.97 / 0.97 / 0.97	0.93 / 0.93 / 0.93 / 0.93	0.89 / 0.89 / 0.89 / 0.89	0.12 / 0.12 / 0.12 / 0.12	0.65 / 0.66 / 0.66 / 0.67	0.07 / 0.07 / 0.07 / 0.07	0.91 / 0.91 / 0.91 / 0.91	0.59 / 0.59 / 0.59 / 0.60	0.26 / 0.26 / 0.26 / 0.26
**Henrietta (1)**									0.87 / 0.87 / 0.86 / 0.86	0.86 / 0.84 / 0.83 / 0.82	0.80 / 0.80 / 0.80 / 0.81	0.97 / 0.97 / 0.97 / 0.97	0.15 / 0.15 / 0.15 / 0.15	0.65 / 0.66 / 0.66 / 0.66	0.98 / 0.99 / 0.99 / 0.99	0.89 / 0.89 / 0.89 / 0.89	0.10 / 0.10 / 0.10 / 0.10	0.80 / 0.78 / 0.77 / 0.75	0.59 / 0.59 / 0.60 / 0.60	0.27 / 0.27 / 0.27 / 0.27
**Henrietta (2)**										0.88 / 0.87 / 0.87 / 0.87	0.77 / 0.77 / 0.77 / 0.76	0.98 / 0.98 / 0.98 / 0.98	0.64 / 0.61 / 0.57 / 0.53	0.74 / 0.74 / 0.75 / 0.75	0.87 / 0.87 / 0.86 / 0.86	0.90 / 0.90 / 0.90 / 0.90	0.72 / 0.69 / 0.67 / 0.64	0.88 / 0.88 / 0.88 / 0.87	0.66 / 0.66 / 0.65 / 0.64	0.28 / 0.28 / 0.28 / 0.28
**Isabel (1)**											0.80 / 0.80 / 0.80 / 0.80	0.97 / 0.97 / 0.97 / 0.97	0.95 / 0.95 / 0.95 / 0.95	0.89 / 0.89 / 0.89 / 0.89	0.13 / 0.13 / 0.13 / 0.13	0.66 / 0.66 / 0.66 / 0.67	0.08 / 0.08 / 0.08 / 0.08	0.86 / 0.85 / 0.85 / 0.84	0.59 / 0.59 / 0.60 / 0.60	0.27 / 0.27 / 0.27 / 0.27
**Isabel (2)**												0.97 / 0.97 / 0.97 / 0.97	0.80 / 0.80 / 0.81 / 0.81	0.87 / 0.87 / 0.87 / 0.87	0.93 / 0.95 / 0.98 / 0.98	0.63 / 0.63 / 0.63 / 0.64	0.88 / 0.90 / 0.92 / 0.94	0.79 / 0.79 / 0.79 / 0.79	0.59 / 0.58 / 0.58 / 0.58	0.28 / 0.28 / 0.28 / 0.29
**Isabel (3)**													0.97 / 0.97 / 0.97 / 0.97	0.98 / 0.98 / 0.98 / 0.98	0.97 / 0.97 / 0.97 / 0.97	0.98 / 0.98 / 0.98 / 0.98	0.97 / 0.97 / 0.97 / 0.97	0.97 / 0.97 / 0.97 / 0.97	0.98 / 0.98 / 0.98 / 0.98	0.97 / 0.96 / 0.95 / 0.93
**Luke (1)**														0.89 / 0.89 / 0.89 / 0.89	0.25 / 0.23 / 0.21 / 0.20	0.66 / 0.66 / 0.66 / 0.67	0.32 / 0.28 / 0.25 / 0.23	0.91 / 0.91 / 0.91 / 0.91	0.59 / 0.60 / 0.60 / 0.60	0.28 / 0.28 / 0.28 / 0.28
**Luke (2)**															0.88 / 0.87 / 0.87 / 0.86	0.94 / 0.94 / 0.94 / 0.95	0.88 / 0.88 / 0.88 / 0.88	0.89 / 0.89 / 0.89 / 0.89	0.95 / 0.97 / 0.99 / 0.98	0.61 / 0.56 / 0.51 / 0.46
**Mittark (1)**																0.56 / 0.54 / 0.53 / 0.52	0.64 / 0.64 / 0.63 / 0.62	0.12 / 0.12 / 0.12 / 0.12	0.52 / 0.52 / 0.52 / 0.53	0.27 / 0.27 / 0.27 / 0.27
**Mittark (3)**																	0.59 / 0.58 / 0.57 / 0.57	0.66 / 0.66 / 0.66 / 0.67	0.91 / 0.87 / 0.83 / 0.80	0.34 / 0.33 / 0.33 / 0.32
**Moffet (1)**																		0.08 / 0.08 / 0.07 / 0.07	0.54 / 0.54 / 0.55 / 0.55	0.27 / 0.27 / 0.27 / 0.27
**Moffet (2)**																			0.59 / 0.59 / 0.59 / 0.60	0.26 / 0.26 / 0.26 / 0.26
**Chip (1)**																				0.37 / 0.38 / 0.38 / 0.38

doi:10.1371/journal.pone.XXXXXXX.YYYY
