# Supplementary material for: Juvenile Osprey Navigation during Trans-Oceanic Migration
Source: PLoS One. 2014 Dec 10;9(12):e114557. doi: 10.1371/journal.pone.0114557 (PMC4262435; doi:10.1371/journal.pone.0114557)
Supplement: Table S2 — Probabilities that paired juvenile osprey migration segments are significantly different in transformed magnetic bicoordinate space. (PDF) [file pone.0114557.s005.pdf]

**Table S2.** Probability matrix for t-tests performed on paired osprey track segments in transformed magnetic coordinate space ($y_T$ *vs.* $z_T$).

| | Bea (2) | Belle (1) | Belle (2) | Belle (3) | Caley (1) | Caley (2) | Caley (3) | Caley (4) | Felix (1) | Felix (2) | Henrietta (1) | Henrietta (2) | Isabel (1) | Isabel (2) | Isabel (3) | Luke (1) | Luke (2) | Mittark (1) | Mittark (2) | Mittark (3) | Moffet (1) | Moffet (2) | Chip (1) | Chip (2) |
|---|---|---|---|---|---|---|---|---|---|---|---|---|---|---|---|---|---|---|---|---|---|---|---|---|
| **Bea (1)** | 0.00 | 0.00 | 0.00 | 0.00 | 0.04 | 0.00 | 0.01 | 0.00 | 0.00 | 0.04 | **0.06** | 0.00 | 0.03 | 0.00 | 0.00 | **0.57** | **0.56** | 0.03 | 0.00 | 0.00 | 0.04 | 0.00 | 0.02 | 0.00 |
| **Bea (2)** | | 0.00 | 0.00 | 0.00 | 0.00 | **0.06** | 0.00 | 0.00 | 0.00 | 0.00 | 0.00 | **0.69** | 0.00 | 0.00 | **0.09** | 0.00 | 0.00 | 0.00 | 0.00 | 0.00 | 0.01 | 0.00 | **0.92** | 0.00 |
| **Belle (1)** | | | 0.00 | 0.00 | 0.00 | 0.00 | 0.00 | 0.00 | 0.00 | 0.00 | 0.00 | 0.00 | 0.00 | 0.00 | 0.00 | 0.00 | 0.00 | 0.00 | 0.00 | 0.00 | **0.22** | 0.00 | 0.00 | 0.00 |
| **Belle (2)** | | | | 0.00 | **0.20** | 0.00 | **0.20** | 0.00 | 0.00 | 0.00 | 0.00 | 0.00 | 0.01 | 0.00 | 0.00 | 0.00 | 0.00 | 0.00 | 0.00 | 0.00 | **0.34** | 0.00 | 0.00 | 0.00 |
| **Belle (3)** | | | | | 0.00 | 0.00 | 0.00 | **0.32** | 0.00 | 0.00 | 0.00 | 0.00 | 0.00 | 0.00 | 0.01 | 0.00 | 0.00 | 0.00 | 0.00 | 0.01 | 0.00 | 0.00 | **0.05** | 0.00 |
| **Caley (1)** | | | | | | 0.00 | **0.80** | 0.00 | 0.00 | 0.02 | 0.00 | 0.00 | **0.31** | 0.00 | 0.00 | 0.01 | **0.09** | 0.00 | 0.00 | 0.00 | **0.17** | 0.00 | 0.01 | 0.00 |
| **Caley (2)** | | | | | | | 0.00 | 0.00 | **0.51** | 0.00 | 0.00 | **0.08** | 0.00 | **0.26** | **0.62** | 0.00 | 0.00 | 0.00 | **0.13** | 0.00 | 0.01 | **0.47** | **0.61** | **0.73** |
| **Caley (3)** | | | | | | | | 0.00 | 0.00 | 0.00 | 0.00 | 0.00 | **0.07** | 0.00 | 0.00 | 0.00 | 0.02 | 0.00 | 0.00 | 0.00 | **0.18** | 0.00 | 0.00 | 0.00 |
| **Caley (4)** | | | | | | | | | 0.00 | 0.00 | 0.00 | 0.00 | 0.00 | 0.00 | 0.01 | 0.00 | 0.00 | 0.00 | 0.00 | 0.00 | 0.00 | 0.00 | **0.05** | 0.00 |
| **Felix (1)** | | | | | | | | | | 0.00 | 0.00 | 0.00 | 0.00 | **0.23** | **0.85** | 0.00 | 0.00 | 0.00 | 0.00 | 0.00 | 0.00 | 0.00 | **0.45** | **0.51** |
| **Felix (2)** | | | | | | | | | | | **0.52** | 0.00 | 0.00 | 0.00 | 0.00 | 0.01 | 0.01 | **0.57** | 0.00 | 0.00 | 0.03 | 0.00 | **0.22** | 0.00 |
| **Henrietta (1)** | | | | | | | | | | | | 0.00 | 0.00 | 0.00 | 0.00 | 0.03 | 0.01 | **0.37** | 0.00 | 0.00 | 0.02 | 0.00 | **0.15** | 0.00 |
| **Henrietta (2)** | | | | | | | | | | | | | 0.00 | 0.00 | **0.11** | 0.00 | 0.00 | 0.00 | 0.00 | 0.00 | 0.01 | 0.01 | **0.96** | 0.00 |
| **Isabel (1)** | | | | | | | | | | | | | | 0.00 | 0.00 | 0.00 | **0.12** | 0.00 | 0.00 | 0.00 | **0.09** | 0.00 | 0.00 | 0.00 |
| **Isabel (2)** | | | | | | | | | | | | | | | **0.88** | 0.00 | 0.00 | 0.00 | **0.41** | 0.00 | 0.01 | 0.00 | **0.37** | **0.16** |
| **Isabel (3)** | | | | | | | | | | | | | | | | 0.00 | 0.00 | 0.00 | **0.69** | 0.04 | 0.01 | **0.32** | **0.46** | **0.73** |
| **Luke (1)** | | | | | | | | | | | | | | | | | **0.18** | 0.01 | 0.00 | 0.00 | 0.03 | 0.00 | 0.03 | 0.00 |
| **Luke (2)** | | | | | | | | | | | | | | | | | | 0.00 | 0.00 | 0.00 | **0.06** | 0.00 | 0.01 | 0.00 |
| **Mittark (1)** | | | | | | | | | | | | | | | | | | | 0.00 | 0.00 | 0.02 | 0.00 | **0.24** | 0.00 |
| **Mittark (2)** | | | | | | | | | | | | | | | | | | | | 0.00 | 0.01 | 0.00 | **0.32** | 0.01 |
| **Mittark (3)** | | | | | | | | | | | | | | | | | | | | | 0.00 | 0.00 | **0.06** | 0.00 |
| **Moffet (1)** | | | | | | | | | | | | | | | | | | | | | | 0.00 | 0.01 | 0.01 |
| **Moffet (2)** | | | | | | | | | | | | | | | | | | | | | | | **0.75** | 0.03 |
| **Chip (1)** | | | | | | | | | | | | | | | | | | | | | | | | **0.52** |

P-values that are not significantly different (α=0.05) are shown in bold. doi:10.1371/journal.pone.XXXXXXX.YYYY
