# Supplementary material for: Juvenile Osprey Navigation during Trans-Oceanic Migration
Source: PLoS One. 2014 Dec 10;9(12):e114557. doi: 10.1371/journal.pone.0114557 (PMC4262435; doi:10.1371/journal.pone.0114557)
Supplement: Table S3 — Probabilities that paired juvenile osprey migration segments are significantly different in Mercator projection geographic bicoordinate space. (PDF) [file pone.0114557.s006.pdf]

Table S3. Probability matrix for t-tests performed on paired osprey track segments in Mercator coordinate space.

	Bea (2)	Belle (1)	Belle (2)	Belle (3)	Caley (1)	Caley (2)	Caley (3)	Caley (4)	Felix (1)	Felix (2)	Henrietta (1)	Henrietta (2)	Isabel (1)	Isabel (2)	Isabel (3)	Luke (1)	Luke (2)	Mittark (1)	Mittark (2)	Mittark (3)	Moffet (1)	Moffet (2)	Chip (1)	Chip (2)
Bea (1)	**0.11**	0.00	**0.36**	0.03	0.00	**0.55**	0.00	0.03	**0.05**	**0.28**	**0.38**	**0.11**	0.00	**0.05**	**0.07**	**0.83**	**0.32**	**0.19**	**0.35**	0.03	0.00	**0.06**	0.00	0.04
Bea (2)		0.00	0.00	0.00	0.00	0.03	0.00	0.00	0.00	**0.94**	0.00	**0.73**	0.00	0.00	**0.06**	**0.92**	0.00	0.00	0.00	0.00	0.00	0.00	0.00	0.00
Belle (1)			0.00	0.00	0.00	0.00	0.00	0.00	0.00	**0.05**	0.00	0.00	0.00	0.00	0.00	**0.63**	0.00	0.00	0.00	0.00	0.00	0.00	0.00	0.00
Belle (2)				0.00	0.00	0.00	0.00	0.00	0.00	0.03	0.00	0.00	0.00	0.00	0.00	**0.69**	**0.68**	0.00	0.00	0.00	0.00	0.00	0.00	0.00
Belle (3)					0.00	0.00	0.00	**0.51**	0.00	**0.44**	0.00	0.00	0.00	0.00	0.00	**0.81**	0.00	0.00	0.00	0.00	0.00	0.00	0.00	0.00
Caley (1)						0.00	0.00	0.00	0.00	**0.08**	0.00	0.00	0.00	0.00	0.00	**0.57**	0.00	0.00	0.00	0.00	0.02	0.00	0.00	0.00
Caley (2)							0.00	0.00	0.00	**0.42**	**0.56**	0.03	0.00	0.00	0.01	**0.92**	**0.05**	**0.11**	**0.48**	0.00	0.00	0.01	0.00	0.00
Caley (3)								0.00	0.00	0.00	0.00	0.00	0.00	0.00	0.00	**0.38**	0.00	0.00	0.00	0.00	0.00	0.00	0.00	0.00
Caley (4)									0.00	**0.47**	0.00	0.00	0.00	0.00	0.00	**0.81**	0.00	0.00	0.00	0.00	0.00	0.00	0.00	0.00
Felix (1)										**0.67**	0.00	0.00	0.00	0.00	**0.71**	**0.87**	0.00	0.00	0.00	0.00	0.00	0.00	0.00	0.00
Felix (2)											**0.51**	**0.92**	0.00	**0.57**	**0.72**	**0.94**	**0.05**	**0.83**	**0.59**	**0.49**	0.04	**0.74**	0.00	**0.56**
Henrietta (1)												0.00	0.00	0.00	0.00	**0.96**	0.02	0.00	**0.69**	0.00	0.00	0.00	0.00	0.00
Henrietta (2)													0.00	0.00	**0.09**	**0.92**	0.00	0.00	0.00	0.00	0.00	0.00	0.00	0.00
Isabel (1)														0.00	0.00	**0.06**	0.00	0.00	0.00	0.00	0.00	0.00	0.04	0.00
Isabel (2)															**0.15**	**0.85**	0.00	0.00	0.00	0.00	0.00	0.00	0.00	**0.60**
Isabel (3)																**0.88**	0.00	0.00	0.00	0.02	0.00	**0.75**	0.00	**0.12**
Luke (1)																	**0.64**	**0.97**	**0.97**	**0.83**	**0.60**	**0.89**	**0.17**	**0.85**
Luke (2)																		0.01	0.02	0.00	0.00	0.00	0.00	0.00
Mittark (1)																			0.04	0.00	0.00	0.00	0.00	0.00
Mittark (2)																				0.00	0.00	0.00	0.00	0.00
Mittark (3)																					0.00	0.00	0.00	0.01
Moffet (1)																						0.00	0.00	0.00
Moffet (2)																							0.00	0.00
Chip (1)																								0.00

P-values that are not significantly different (α=0.05) are shown in bold. doi:10.1371/journal.pone.XXXXXXX.YYYY
