# Supplementary material for: Juvenile Osprey Navigation during Trans-Oceanic Migration
Source: PLoS One. 2014 Dec 10;9(12):e114557. doi: 10.1371/journal.pone.0114557 (PMC4262435; doi:10.1371/journal.pone.0114557)
Supplement: File S1 — This file includes supporting text explaining how to use the freely downloadable magnetic models applied in this study. (DOCX) [file pone.0114557.s007.docx]

The Linux-based Enhanced Magnetic Model (i.e. EMM) software is run from the command-line interface with the ability to batch process multiple coordinate locations compiled in a delimited text file with a single line of code, such as:

X:\Geomag>emm_sph_file_windows.exe f Ospreysin.txt Ospreysout.txt

Where, “X:\Geomag>” denotes the current computer drive (X:\) and directory (Geomag) that hosts both the EMM software package and the text format data file that will be called; “>” denotes the end of the command prompt; “emm_sph_file_windows.exe” is an executable command entered by the user that calls the spherical harmonic approach software in a Windows environment; “f” is a switching function that tells the software to call the user-defined file “Ospreysin.txt” for batch processing; “Ospreysout.txt” is the user-defined output data file that will be created and stored in the current drive\directory. A similar command-line operated software package, Geomag7.0, is available for WMM coordinate calculations from N.O.A.A.’s geomagnetism web-portal (http://ngdc.noaa.gov/geomag/), as well as a variety of utility programs including ones capable of estimating the diel magnetic field variation and positions of the sun and moon at user-defined geographic coordinate locations and times. We mention these analytical tools as they are widely utilised by geophysicists, but are perhaps less well known to movement ecologists.
